# A Large-Scale Genome-Wide Association Study in U.S. Holstein Cattle

**DOI:** 10.3389/fgene.2019.00412

**Published:** 2019-05-14

**Authors:** Jicai Jiang, Li Ma, Dzianis Prakapenka, Paul M. VanRaden, John B. Cole, Yang Da

**Affiliations:** ^1^Department of Animal and Avian Sciences, University of Maryland, College Park, MD, United States; ^2^Department of Animal Science, University of Minnesota, Saint Paul, MN, United States; ^3^Animal Genomics and Improvement Laboratory, USDA-ARS, Beltsville, MD, United States

**Keywords:** GWAS, dairy cattle, milk production, fertility, somatic cell score

## Abstract

Genome-wide association study (GWAS) is a powerful approach to identify genomic regions and genetic variants associated with phenotypes. However, only limited mutual confirmation from different studies is available. We conducted a large-scale GWAS using 294,079 first-lactation Holstein cows and identified new additive and dominance effects on five production traits, three fertility traits, and somatic cell score. Four chromosomes had the most significant SNP effects on the five production traits, a Chr14 region containing *DGAT1* mostly had positive effects on fat yield and negative effects on milk and protein yields, the 88.07–89.60 Mb region of Chr06 with *SLC4A4, GC, NPFFR2*, and *ADAMTS3* for milk and protein yields, the 30.03–36.67 Mb region of Chr20 with *C6* and *GHR* for milk yield, and the 88.19–88.88 Mb region with *ABCC9* as well as the 91.13–94.62 Mb region of Chr05 with *PLEKHA5, MGST1, SLC15A5*, and *EPS8* for fat yield. For fertility traits, the SNP in *GC* of Chr06, and the SNPs in the 65.02–69.43 Mb region of Chr01 with *COX17, ILDR1*, and *KALRN* had the most significant effects for daughter pregnancy rate and cow conception rate, whereas SNPs in *AFF1* of Chr06, the 47.54–52.79 Mb region of Chr07, *TSPAN4* of Chr29, and *NPAS1* of Chr18 had the most significant effects for heifer conception rate. For somatic cell score, *GC* of Chr06 and *PRLR* of Chr20 had the most significant effects. A small number of dominance effects were detected for the production traits with far lower statistical significance than the additive effects and for fertility traits with similar statistical significance as the additive effects. Analysis of allelic effects revealed the presence of uni-allelic, asymmetric, and symmetric SNP effects and found the previously reported *DGAT1* antagonism was an extreme antagonistic pleiotropy between fat yield and milk and protein yields among all SNPs in this study.

## Introduction

The discovery of quantitative trait loci (QTL) is an important step to identify and understand genetic variants associated with economically important phenotypes, and genome-wide association study (GWAS) has become a widely used approach for identifying QTL and genome regions associated with phenotypes. GWAS in several dairy cattle breeds have reported a large number of QTL effects on dairy traits (Bolormaa et al., 2010; Pryce et al., 2010, 2014; Cole et al., 2011; Guo et al., 2012; Ma et al., 2012; Rothammer et al., 2013; Raven et al., 2014; Littlejohn et al., 2016; Jiang et al., 2017; Sanchez et al., 2017; Bouwman et al., 2018; Weller et al., 2018). However, the number of confirmed QTL effects across studies is low (Ma et al., 2018; Weller et al., 2018), and only limited understanding of the genetic mechanism of the QTL effects is available. The *DGAT1* gene was widely confirmed to have the most significant effects for milk production (Grisart et al., 2002; Spelman et al., 2002; Schennink et al., 2007; Cole et al., 2011; Ma et al., 2012; Jiang et al., 2017), and previously was shown to have antagonistic pleiotropy effects between fat yield and milk and protein yields based on candidate causal variants (Thaller et al., 2003) or causal alleles (da Silva et al., 2010). However, it was unknown whether the *DGAT1* antagonism was the strongest of its kind on the genome or how far the antagonism extends around *DGAT1*. To build consensus about QTL effects affecting dairy traits and to understand the genetic mechanisms of significant SNP effects, additional studies are needed. A powerful approach to build consensus on QTL effects is the use of large samples for GWAS (Bouwman et al., 2018; Dadaev et al., 2018; Gurevitch et al., 2018; Nagel et al., 2018; Yengo et al., 2018). The U.S. Holstein cattle have uniquely large sample sizes (VanRaden, 2016; Wiggans et al., 2017) and provides an opportunity to identify SNPs associated with dairy traits using GWAS. The purpose of this study was to identify SNPs associated with nine dairy traits using a large-scale GWAS combined with the analysis of allelic effects in Holstein cattle to provide a large-sample perspective of dairy QTL effects.

## Material and Methods

### Holstein Populations and Genotyping Data

The sample for GWAS analysis contained 294,079 first lactation Holstein cows with phenotypic observations for five milk production traits (milk, fat and protein yields, and fat and protein percentages), three fertility traits (daughter pregnancy rate, cow conception rate, and heifer conception rate), and somatic cell score. Daughter pregnancy rate is the percentage of cows that become pregnant during each 21-d period, and cow and heifer conception rate each is defined as percentage pregnancy at each service (Ma et al., 2018). The number of phenotypic observations ranged from 294,079 for milk yield to 186,188 for cow conception rate (Table S1). The 294,079 cows had SNP genotypes of 60,671 SNPS (60K) imputed from 18 SNP chips with 2,710 to 60,671 original SNPs (Table S2). The imputed 60K SNPs were from the dairy genomic evaluation at the Council of Dairy Cattle Breeding (CDCB) and the Animal Genomics and Improvement Laboratory at U.S. Department of Agriculture (USDA). Of the 294,079 cows for the GWAS, 98.4% were born between 2006 and 2015 (Table S3).

### GWAS Analysis

The GWAS analysis used two methods, an approximate generalized least squares (AGLS) analysis, and a Bayesian linear mixed model implemented by the BOLT-LMM program. The BOLT-LMM method accounts for population stratification and cryptic relatedness using “leave-one-chromosome-out” genomic relationships, overcomes the computing difficulties of other mixed model methods such as GCTA (Yang et al., 2011) and EMMAX (Kang et al., 2010) that use SNP relationships for sample stratification correction (Loh et al., 2015; Loh, 2018), and was able to analyze a large human sample with ~700,000 individuals for GWAS (Yengo et al., 2018). However, BOLT-LMM tests additive effects only. The AGLS method is original to this study with capability to test both additive and dominance effects, estimate additive, allelic and dominance effects, and estimate allele and genotypic frequencies. Results in this study showed AGLS and BOLT-LMM virtually identified the same sets of additive SNP effects with only minor differences in effect rankings, indicating that AGLS had similar efficiency as BOLT-LMM for sample stratification correction. The main difference was that BOLT-LMM had much smaller *p*-values and much larger effect sizes than those of AGLS. As an extreme case, the most significant SNP effect for fat percentage had a *p*-value of 3.7(10^−10,871^) from BOLT-LMM or 6.2(10^−5,150^) from AGLS, and the effect size from BOLT-LMM was 79% larger than that from AGLS. As a result of the much smaller *p*-values from BOLT-LMM than from AGLS, BOLT-LMM had 46% more significant additive SNP effects than AGLS. Therefore, AGLS was more conservative in declaring significance and likely was more realistic for the estimated effect sizes than BOLT-LMM, with additional benefit of testing dominance effects and estimating allelic and dominance effects that were unavailable from BOLT-LMM.

The AGLS method combines the least squares (LS) tests implemented by EPISNP1mpi (Ma et al., 2008; Weeks et al., 2016) with the estimated breeding values from routine genetic evaluation using the entire U.S. Holstein population. The statistical model was

(1) $$\begin{array}{l} \text{y}=\;\mu {\text{I }+\text{ X}}_{\text{g}}\text{g }+\text{ Za }+\text{ e }=\text{ Xb }+\text{ Za }+\text{ e} \end{array}$$

where **y** = column vector of phenotypic deviation after removing fixed non-genetic effects such as heard-year-season (termed as “yield deviation” for any trait) using a standard procedure for the CDCB/USDA genetic and genomic evaluation; μ = common mean; **I** = identity matrix; $\text{g}={\left(g_{11},g_{12},g_{22}\right)}^{\prime }$ = column vector of genotypic values of the three SNP genotypes *A*_1_*A*_1_, *A*_1_*A*_2_, and *A*_2_*A*_2_; **X**_g_ = model matrix of **g**; **b** = (μ, **g**′)′, **X** = (**I**, **X**_g_); **a** = column vector of additive polygenic values; **Z** = model matrix of **a** = identity matrix if each individual has one observation; and **e** = random residuals. The first and second moments of Equation 1 are: E(**y**) = **Xb**, and var(**y**) = **V** = **ZGZ**′ + **R** = $\;\sigma {\;}_{\text{a}}^{\text{2}}$**ZAZ**′ + $\;\sigma {\;}_{\text{e}}^{\text{2}}$**I**, where $\;\sigma {\;}_{\text{a}}^{\text{2}}$ = additive variance, **A** = additive relationship matrix, and $\;\sigma {\;}_{\text{e}}^{\text{2}}$ = residual variance. The problem of estimating the **b** vector in Equation 1 is the requirement of inverting the **V** if the generalized least squares (GLS) equations are used, or inverting the **A** matrix if the mixed model equations (MME) (Henderson, 1984) are used. However, both **V** and **A** cannot be inverted for our sample size. The first approximation of the AGLS method is to use existing estimates of **a** from routine genetic evaluation so that inverting **V** or **A** is no longer required for estimating **b**. This approximation is based on the following results:

(2) $$\begin{array}{lll} \hat{\text{b}} & = & {\left({\text{X}}^{\prime }{\text{V}}^{-1}\text{X}\right)}^{-}{\text{X}}^{\prime }{\text{V}}^{-1}\text{y} \end{array}$$

(3) $$\begin{array}{ll} \hat{\text{b}}\;= & {\left({\text{X}}^{\prime }{\text{R}}^{-1}\text{X}\right)}^{-}\left({\text{X}}^{\prime }{\text{R}}^{-1}\text{y}-{\text{X}}^{\prime }{\text{R}}^{-1}\text{Z}\hat{\text{a}}\right)={\left({\text{X}}^{\prime }\text{X}\right)}^{-}{\text{X}}^{\prime }\left(\text{y}-\text{Z}\hat{\text{a}}\right)\\ \;= & {\left({\text{X}}^{\prime }\text{X}\right)}^{-}{\text{X}}^{\prime }{\text{y}}_{\text{*}} \end{array}$$

where ${\text{y}}_{*}=\text{y}-\text{Z}\hat{\text{a}}$, and where $\hat{\text{a}}$ = the best linear unbiased prediction (BLUP) of **a**. Equation 2 is the GLS solution and Equation 3 is the MME solution of **b**. These two equations yield identical results and the $\hat{\text{b}}$ from either equation is termed as the best linear unbiased estimator (BLUE) (Henderson, 1984). Equations 2, 3 have two important messages. First, the GLS solution or BLUE of Equation 2 in fact has the same formula as the LS solution of Equation 3 if the residual variance-covariance matrix has the structure of **R** = $\;\sigma {\;}_{\text{e}}^{\text{2}}$**I** and the BLUP of **a** (denoted by $\hat{\text{a}}$) is removed from the phenotypic observations. Second, the GLS solution or BLUE of Equation 2 in fact removes $\hat{\text{a}}$ from the phenotypic observations as shown by the equivalence between Equations 2 and 3 even though Equation 2 does not show the removal of $\hat{\text{a}}$ explicitly. If $\hat{\text{a}}$ is known, the LS version of BLUE given by Equation 3 is computationally efficient relative to the GLS of Equation 2 requiring the **V** inverse, or the joint MME solutions of $\hat{\text{b}}$ and $\hat{\text{a}}$ requiring the **A** inverse. Therefore, we used estimates of **a** ($\tilde{\text{a}}$) from routine genetic evaluation as an approximation of $\hat{\text{a}}$ in Equation 3, i.e.,

(4) $$\begin{array}{l} \hat{\text{b}}={\left({\text{X}}^{\prime }\text{X}\right)}^{-}{\text{X}}^{\prime }\left(\text{y}-\text{Z}\tilde{\text{a}}\right)={\left({\text{X}}^{\prime }\text{X}\right)}^{-}{\text{X}}^{\prime }{\text{y}}^{*} \end{array}$$

where ${\text{y}}^{*}=\text{y}-\text{Z}\tilde{\text{a}}$, and where $\tilde{\text{a}}$ = column vector of 2(PTA), PTA = predicted transmission ability from routine genetic evaluation. Equation 4 achieves the benefit of sample stratification correction from mixed models without the computing difficulty of inverting **V** or **A**, as shown by the nearly identical SNP effects detected by both BOLT-LMM and AGLS. Moreover, the $\tilde{\text{a}}$ from routine genetic evaluation in Equation 4 should be more accurate than the $\hat{\text{a}}$ in Equation 3, because the sample size for $\tilde{\text{a}}$ generally was much larger than the sample size of a GWAS study. For example, the August 2017 Holstein genetic evaluation used 87,729,358 Holstein cows whereas our GWAS used 294,079 cows. Consequently, the approximate $\hat{\text{g}}$ in the $\hat{\text{b}}$ vector of Equation 4 should be more accurate than the $\hat{\text{g}}$ in the $\hat{\text{b}}$ vector of Equation 3. The second approximation of the AGLS approach is the *t*-test using the LS rather than the GLS formula of the t-statistic to avoid using the **V** inverse in the GLS formula. The significance tests for additive and dominance SNP effects used the *t*-tests of the additive and dominance contrasts of the estimated SNP genotypic values (Mao et al., 2007; Ma et al., 2012). The *t*-statistic of the AGLS was calculated as:

(5) $$\begin{array}{l} {\text{t}}_{\text{j}}\text{=}\frac{\;|\;{\text{L}}_{\text{j}}|}{\sqrt{\text{var(}{\text{L}}_{\text{j}}\text{)}}}=\frac{\left|{\text{s}}_{\text{j}}^{}\hat{\text{g}}\right|}{\text{v}\sqrt{{\text{s}}_{\text{j}}^{}{\left({\text{X}}_{}^{{}^{\prime }}{\text{X}}_{}\right)}_{\text{gg}}^{-}{\text{s}}_{\text{j}}^{{}^{\prime }}}},\text{j}=\text{a,d} \end{array}$$

where L_j_ = additive or dominance contrast, $\sqrt{\text{var(}{\text{L}}_{\text{j}}\text{)}}$ = standard deviation of the additive or dominance contrast, **s**_a_ = additive contrast coefficients = (P_11_/p_1_,0.5P_12_(p_2_−p_1_)/(p_1_p_2_), –P_22_/p_2_); **s**_d_ = dominance contrast coefficients = (−0.5, 1, −0.5); ${\text{v}}^{2}\text{=}\left(y-X\hat{b}\right)\text{'}\left(y-x\hat{b}\right)\text{/}\left(\text{n}-\text{k}\right)$ = estimated residual variance; $\hat{\text{g}}$ = column vector of the AGLS estimates of the three SNP genotypic effects of g_11_, g_12_, and g_22_ from Equation 4; ${\left({\text{X}}_{}^{{}^{\prime }}{\text{X}}_{}\right)}_{\text{gg}}^{-}$ = submatrix of ${\left({\text{X}}_{}^{{}^{\prime }}{\text{X}}_{}\right)}_{}^{-}$ corresponding to $\hat{\text{g}}$; and where p_1_ = frequency of *A*_1_ allele, p_2_ = frequency of *A*_2_ allele of the SNP, P_11_ = frequency of *A*_1_*A*_1_ genotype, P_12_ = frequency of *A*_1_*A*_2_ genotype, P_22_ = frequency of *A*_2_*A*_2_ genotype, n = number of observations, and k = rank of **X**. The formula of **s**_a_ defined above allows Hardy-Weinberg disequilibrium (Mao et al., 2007), and simplifies to (p_1_, p_2_−p_1_, –p_2_) under Hardy-Weinberg equilibrium.

In addition to being a computationally efficient method for sample stratification correction for large samples, the AGLS method implemented by EPSNPmpi (Ma et al., 2008; Weeks et al., 2016) offers tests and estimates unavailable from BOLT-LMM, including dominance test, estimates of allelic and dominance effects, and estimates of allele and genotypic frequencies of each SNP. Additive effects of each SNP were estimated using three measures, the average effect of gene substitution, allelic mean, and allelic effect of each allele based on quantitative genetics definitions (Falconer and Mackay, 1996; Mao et al., 2007; Da, 2015). The allelic mean (μ_i_), the population mean of all genotypic values of the SNP (μ), the allelic effect (a_i_), and the average effect of gene substitution of the SNP (α) are:

(6) $$\begin{array}{l} {\;\mu \;}_{1}={\text{P}}_{\text{11.1}}{\text{g}}_{11}\text{+0.5}{\text{P}}_{\text{12.1}}{\text{g}}_{12} \end{array}$$

(7) $$\begin{array}{l} {\;\mu \;}_{2}=\text{0.5}{\text{P}}_{\text{12.2}}{\text{g}}_{12}\text{+}{\text{P}}_{\text{22.2}}{\text{g}}_{22} \end{array}$$

(8) $$\begin{array}{l} \;\mu \;=\sum_{\text{i=1}}^{\text{2}}{\text{p}}_{\text{i}}{\;\mu \;}_{\text{i}} \end{array}$$

(9) $$\begin{array}{l} {\text{a}}_{\text{i}}={\;\mu \;}_{\text{i}}-\;\mu ,\text{ i}=1,2 \end{array}$$

(10) $$\begin{array}{l} \;\alpha ={\text{L}}_{\text{a}}={\text{s}}_{\text{a}}^{}\hat{\text{g}}\text{=}{\text{a}}_{1}-{\text{a}}_{2}={\;\mu \;}_{\text{1}}-{\;\mu \;}_{2} \end{array}$$

where P_11.1_ = P_11_/p_1_, P_12.1_ = P_12_/p_1_, P_12.2_ = P_12_/p_2_, and P_22.2_ = P_22_/p_2_. The additive effect measured by the average effect of gene substitution of Equation 10 is the distance between the two allelic means or effects of the same SNP, and is the fundamental measure for detecting SNP additive effects as shown by the t-statistic of Equation 5. The allelic effects defined by Equation 9 provide an understanding of the effect size and direction of each allele, but is not comparable across SNPs because the allelic effect is affected by the genotypic mean of the SNP defined by Equation 8. To compare allelic effects across SNPs, we replaced the SNP genotypic mean (μ) in Equation 9 with the average of all SNP genotypic means (μ_all_), i.e.,

(11) $$\begin{array}{l} {\text{a}}_{\text{i}}\text{=}{\;\mu \;}_{\text{i}}-{\;\mu \;}_{\text{all}}\;i=1,2, \end{array}$$

Dominance effect of each SNP was estimated as the dominance contrast in Equation 5, i.e.,

(12) $$\begin{array}{l} \;\delta \;={\text{L}}_{\text{d}}={\text{d}}_{\text{12}}-\text{(}{\text{d}}_{\text{11}}\text{+}{\text{d}}_{\text{22}}\text{)}/2={\text{g}}_{\text{12}}-\text{(}{\text{g}}_{\text{11}}+{\text{g}}_{\text{22}})/2 \end{array}$$

where d_ij_ = dominance deviation of the *A*_i_*A*_j_ SNP genotype, and g_ij_ = the AGLS estimates of SNP genotypic value from Equation 4, i, j = 1, 2.

The *t*-tests of additive and dominance effects of each SNP as well as the estimation of each allelic effect and each genotypic value of a SNP were implemented by EPISNPmpi (Ma et al., 2008; Weeks et al., 2016). A limitation in EPISNPmpi causes a *p*-value < 10^−308^ to be printed as “0”. For such *p*-values, we used empirical log_10_(1/p) values as a power function of the observed *t*-values based on the empirical formula of log_10_(1/p) = 0.2416(t^1.9713^), and the empirical log_10_(1/p) values had a 100% correlation with the observed log_10_(1/p) values (Figure S1). The *t*-test of Equation 5 for additive effects accounts for variations associated with allele frequencies and none of the SNPs with rare alleles was among the most significant SNPs for any trait in this study. Generally, the statistical significance of additive effects represented by the *t*-value of Equation 5 decreases as the allele frequencies deviate further away from equal allele frequencies. However, the allelic effects of Equations 9, 11 do not account for variations associated with allele frequencies and many rare alleles with small frequencies had large effects. Therefore, a minor allele frequency (MAF) of 0.05 was required for reporting SNP effect size and direction and 57,067 SNPs satisfied this requirement. For 57,067 SNPs and nine traits, the Bonferroni correction with 0.05 genome-wide false positives is 10^−7^. Nearly all figures were produced using SNPEVG2 in the SNPEVG package (Wang et al., 2012). The SNP positions are those from the UMD 3.1 cattle genome assembly.

## Results and Discussion

### Overview of GWAS Results

The GWAS identified a large number of SNP effects exceeding the statistical significance with the Bonferroni correction for 5% genome-wide false positives (*p* < 10^−7^), 61,062 SNP effects from AGLS and 89,457 SNP effects from BOLT-LMM for the nine dairy traits (Table S4). Majority of these SNPs effects, 58,207 of the 61,602 effects (95%) from AGLS or 84,072 of the 89,457 effects (94%) from BOLT-LMM, were those for the five milk production traits: milk, fat and protein yields, and fat and protein percentages. The method of BOLT-LMM (Loh et al., 2015; Loh, 2018) and the method of AGLS in this study had virtually identical Manhattan plots of *p*-values (Figure S2). These methods virtually identified the same set of highly significant additive effects with minor differences in the rankings of statistical significance (Tables 1–**4**) although the differences in effect ranking between these two methods became larger for less significant SNP effects (Table S5; Figure S3). For the top 200 most significant effects, BOLT-LMM consistently had larger effect sizes than the corresponding AGLS effect size for the five production traits and somatic cell score, but had similar effect sizes as AGLS for the three fertility traits (Figure S3). The correlation between effect rankings of all 60,671 SNPs between these two methods was in the range 0.47 for heifer conception rate to 0.76 for fat percentage. BOLT-LMM generally had much smaller *p*-values than AGLS. For the extreme case of the most significant SNP effect for fat percentage, the *p*-value was 3.7(10^−10,871^) from BOLT-LMM and was 6.2(10^−5,150^) from AGLS. Consequently, BOLT-LMM had 46% more significant SNP effects than AGLS, i.e., 89,457 additive SNP effects from BOLT-LMM and 61,062 additive SNP effects from AGLS with *p* < 10^−7^ (Table S4). BOLT-LMM also had much larger effect sizes than those from AGLS. For the sample of the SNP effect on milk yield for SNP *rs109421300* located in *DGAT1*, the effect size was −248.13 kg from AGLS and was −445.05 kg from BOLT-LMM (Table 1). The AGLS effect size of −248.13 kg was closer to those of previous reports for the effect sizes of the causal variant in *DGAT1*, −180 kg in German Holsteins (Thaller et al., 2003) and −81 kg in U.S. Holsteins (da Silva et al., 2010). Therefore, the effect sizes from AGLS likely were more realistic than the effect sizes from BOLT-LMM, noting that inflated effect size necessarily inflates the statistical significance (reduces the *p*-value) of the SNP. Given this comparison for statistical significance and effect size between BOLT-LMM and AGLS, the AGLS' ability to test dominance effects and estimate allelic and dominance effects, the discussion henceforth mostly uses the AGLS results. The number of dominance SNP effects with *p* < 10^−7^ was 494 for production and fertility traits, about 0.8% of the number of additive effects detected by AGLS, and somatic cell score had no significant dominance effect. The larger number of significant SNP effects of the five production traits than the three fertility traits and somatic cell score was consistent with the fact that the production traits had higher heritabilities than the fertility traits and somatic cell score (Schopen et al., 2009; Jiang et al., 2017).

**Table 1:** Significant additive effects of milk, fat, and protein yields.

	**AGLS**							**BOLT-LMM**		
**SNP**	**Chr**	**Position (bp)**	**Candidate gene**	**Effect (α, kg)**	***t*-value**	***p*-value**	**Rank**	**Effect (β, kg)**	***p*-value**	**Rank**
**MILK YIELD**										
rs109421300	14	1801116	*DGAT1*	−248.13	61.81	1.0E-820^a^	1	−445.05	9.4E-1907	1
rs135549651	14	1967325	*SMPD5*	223.79	57.45	9.5E-710^a^	2	−395.74	4.8E-1593	3
rs109146371	14	1651311	*PPP1R16A*	−213.34	55.06	7.4E-653^a^	3	−392.86	5.2E-1597	2
rs109350371	14	2054457	*PLEC* (u)	−210.23	54.37	7.2E-637^a^	4	−376.92	2.1E-1490	4
rs109558046	14	2909929	*ARC-ADGRB1*	145.29	42.71	1.0E-396^a^	5			6
rs109752439	14	1489496	*ZNF34* (u)	145.00	41.05	1.0E-347^a^	6			5
rs110527224	6	88592295	*GC* (u)	96.49	29.91	1.2E-231	32	−241.13	4.4E-285	49
rs137147462	6	88887995	*GC* (d)	104.43	28.08	2.6E-173	44	139.70	1.8E-259	62
rs110694875	6	89139865	*ADAMTS3* (u)	−96.16	28.08	2.7E-173	45	147.33	1.8E-285	48
rs109901151	6	88494442	*SLC4A4*	92.14	26.76	1.4E-157	52	−143.44	8.2E-276	56
rs41938455	20	33354480	*C6*	136.60	26.60	9.3E-156	53	227.08	1.3E-283	51
rs137431035	20	33824992	*PTGER4* (d)	−137.47	26.57	2.3E-155	54	222.77	3.1E-281	52
rs41573457	20	30036600	*MRPS30* (u)	120.01	24.53	9.3E-133	65	209.05	2.5E-279	54
rs110914335	14	2570165	*LY6H* (d)	−107.26	21.56	5.0E-103	94	161.29	7.1E-166	135
rs110482506	20	32030332	*GHR*	82.31	21.52	1.1E-102	96	142.67	2.1E-211	93
**FAT YIELD**										
rs109421300	14	1801116	*DGAT1*	6.26	41.50	9.0E-374^a^	1	13.15	5.6E-1124	1
rs109146371	14	1651311	*PPP1R16A*	5.49	37.60	4.8E-308	2	11.47	5.1E-905	2
rs109350371	14	2054457	*PLEC* (u)	5.26	36.00	5.0E-283	3	10.84	4.9E-833	3
rs135549651	14	1967325	*SMPD5*	−5.08	34.70	3.7E-263	4	11.04	2.3E-849	4
rs109350371	14	2084067	*LOC786966*	−4.26	31.20	9.8E-214	5	−8.12	5.1E-532	6
rs109558046	14	2909929	*ARC-ADGRB1*	−3.81	29.80	3.1E-194	6	8.39	1.1E-622	5
rs110825637	5	93995487	*MGST1-SLC15A5*	3.37	26.30	6.4E-152	12	5.70	6.5E-295	37
rs137735153	5	91136990	*PLEKHA5*	−3.05	22.70	1.6E-113	27	−4.70	1.1E-184	64
rs42718234	5	88680972	*ABCC9*	−3.31	21.20	1.3E-99	34	−5.64	4.4E-195	60
rs42406616	5	88702470	*ABCC9*	−3.12	20.50	4.6E-93	37	5.59	6.2E-203	56
**PROTEIN YIELD**										
rs109421300	14	1801116	*DGAT1*	−4.50	41.30	3.5E-371^a^	1	−7.60	1.9E-659	1
rs135549651	14	1967325	*SMPD5*	4.16	38.40	8.4E-320^a^	2	−6.84	5.9E-572	2
rs109146371	14	1651311	*PPP1R16A*	−3.86	37.60	4.8E-308	3	−6.76	4.7E-552	3
rs109350371	14	2054457	*PLEC* (u)	−3.82	36.00	5.0E-283	4	−6.53	6.2E-531	4
rs109558046	14	2909929	*ARC-ADGRB1*	2.72	28.80	0.3E-181	5	−4.17	3.8E-276	6
rs109558046	14	1489496	*ZNF34* (u)	2.69	27.94	0.1E-164	6	−4.79	7.3E-340	5
rs110694875	6	89139865	*ADAMTS3* (u)	−2.27	22.40	2.7E-173	13	3.41	1.8E-285	18
rs109901151	6	88494442	*SLC4A4*	2.22	23.20	0.6E-126	16	3.45	5.5E-187	16
rs137147462	6	88887995	*GC* (d)	2.13	28.08	0.3E-118	21	3.06	5.0E-150	26
rs110478571	5	106367181	*CCND2* (u)	−2.05	19.10	4.9E-81	48	−2.97	6.4E-111	56
rs41257416	5	105870613	*NDUFA9*^b^	−1.92	18.30	8.5E-75	55	−2.72	2.5E-102	62
rs110000229	5	105804923	*GALNT8*^b^	1.80	17.70	6.0E-70	59	−2.79	1.1E-102	61
rs110914335	14	2570165	*LY6H* (d)	−2.08	15.10	1.8E-51	113	2.99	1.8E-68	148

a *This is the empirical p-value based on the observed t-value using the formula of $\text{lo}{\text{g}}_{10}\text{(1/p)=0}\text{.2416(}{\text{t}}^{\text{1}\text{.9713}}\text{)}$ (Figure S1), because the observed p-value was printed as “0” when the t-value is too large due to a limitation in the EPISNPmpi program*.

b These two genes are also highly significant for milk yield (Table S5). “u” indicates the SNP is upstream of the gene, and “d” indicates the SNP is downstream of the gene. “rank” is the rank of the statistical significance. “effect” is the average effect of gene substitution or the difference between allelic effects of “allele 1” and “allele 2.”

The understanding of a large number of SNP effects necessarily will be a long process. Therefore, this article only reports a subset of the SNP effects exceeding the Bonfferoni significance based on mutual confirmation between AGLS and BOLT-LMM. For additive effects of the five milk production traits, the top 1% significant effects from AGLS (570 effects per trait) were selected, and those effects were further filtered by the requirement that the reported effects also were among the top 1% effects by BOLT-LMM. For the low-heritability traits of somatic cell score, daughter pregnancy rate, and cow conception rate (Jiang et al., 2017), the top 200 effects from AGLS were selected and those effects were further filtered by the requirement that the reported effects also were among the top 200 effects by BOLT-LMM. Heifer conception rate had only 15 additive effects exceeding the Bonferroni significance and we report eight of those effects ranked high by both methods. For dominance effects, all effects exceeding the Bonferroni significance are reported because BOLT-LMM does not test dominance effects and because only limited dominance results were available for comparison. In total, this study reports 2617 additive SNP effects involving 1472 SNPs (Table S5) and 494 dominance SNP effects involving 354 SNPs (Table S6) for nine dairy traits. Given the mutual confirmation between AGLS and BOLT-LMM and the large sample size of 294,079 cow, the 2617 additive SNP effects reported in this study should be high confidence SNP effects. In addition to statistical significance and SNP effect size measured as the difference between the two allelic effects of the SNP, we also analyzed the effect size and direction of each allele of a significant SNP, and the allelic analysis provided valuable insights into the genetic mechanism and practical impact of each significant SNP. Since Table S5 has detailed information for all 2617 additive effects, Tables 1–**4** for additive effects only summarizes the top six effects from AGLS and BOLT-LMM along with a few SNPs with large allelic effects (positive or negative) for each trait. Global graphical comparison between statistical significance of each SNP and effect size and direction of the allelic effects of the SNP is shown in Figure S4. The primary purpose of GWAS is to identify candidate genes and chromosome regions associated with phenotypes. For this purpose, the figures and tables in this study show or list genes implicated by the SNP effects.

### Additive SNP Effects of Production Traits

**Figure 1:**
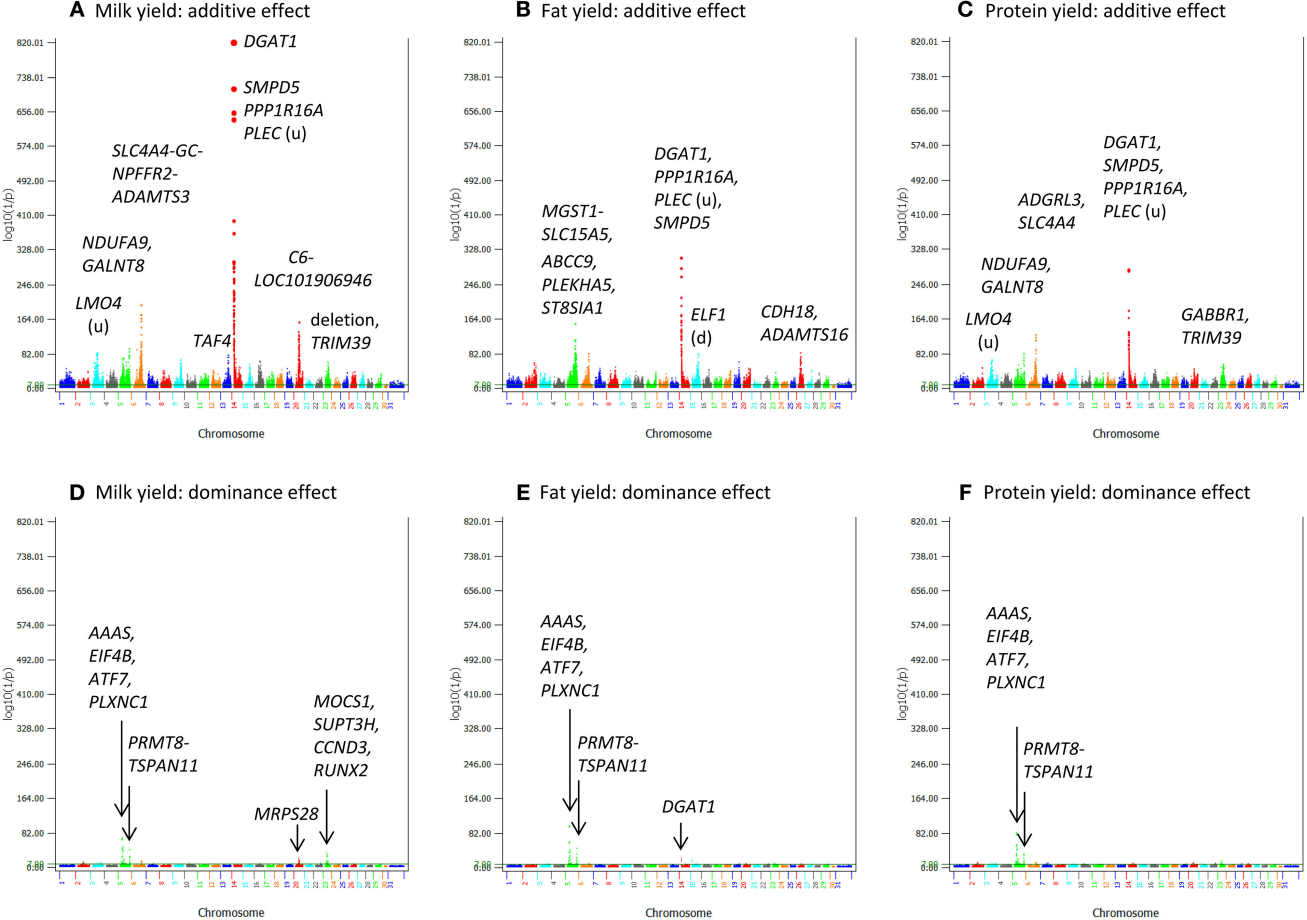
Statistical significance of additive and dominance SNP effects for yield traits. **(A)** Additive effects of milk yield. **(B)** Additive effects of fat yield. **(C)** Additive effects of protein yield. **(D)** Dominance effects of milk yield. **(E)** Dominance effects of fat yield. **(F)** Dominance effects of protein yield. The horizontal line at log10(1/p) = 7 is the threshold for statistical significance of 5% genome-wide false positives with the Bonferroni correction.

**Figure 2:**
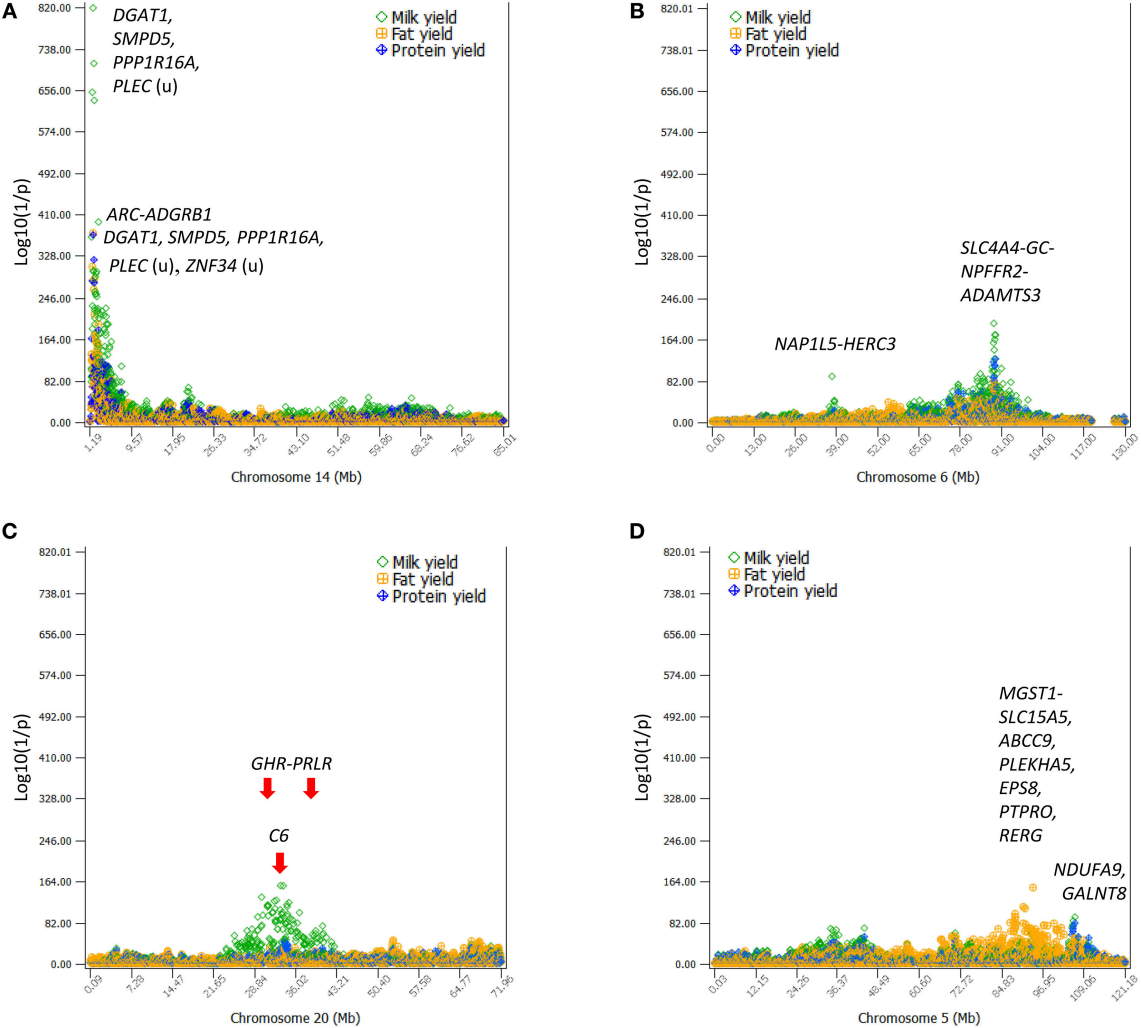
The four chromosomes with the most significant additive effects on milk, fat, and protein yields. **(A)** *DGAT1* of Chr14 and its surrounding region ~5 Mb in size had the most significant effects on all yield traits. **(B)** A 10-Mb region of Chr06 had highly significant SNP effects on all yield traits with peak effects in the *SLC4A4-GC*-*NPFFR2*-*ADAMTS3* region. **(C)** A large 19-Mb region of Chr20 had highly significant SNP effects on milk yield. The *GHR-PRLR* region had a high concentration of significant effects and the *C6* had the peak SNP effect. **(D)** A 9-Mb region of Chr05 had highly significant SNP effects on fat yield with peak effects in the *ABCC9-RERG* region. The horizontal line at log10(1/p) = 7 is the threshold for statistical significance of 5% genome-wide false positives with the Bonferroni correction.

Production traits had the largest number of significant additive SNP effects, with 11,856, 9803, 9984, 11,349, and 15,215 significant additive effects for milk yield, fat yield, protein yield, fat percentage, and protein percentage, respectively (Table S4). Four chromosomes, Chr14, Chr06, Chr20, and Chr05, had the most significant additive SNP effects for the yield traits (Figures 1, 2). Specific regions with significant effects were the 1.42–5.49 Mb region of Chr14, the 88.07–89.60 Mb region of Chr06, the 30.03–36.67 Mb region of Chr20, and the 88.19–88.88 Mb and 91.13–94.62 Mb regions of Chr05 (Figure 2).

**Figure 3:**
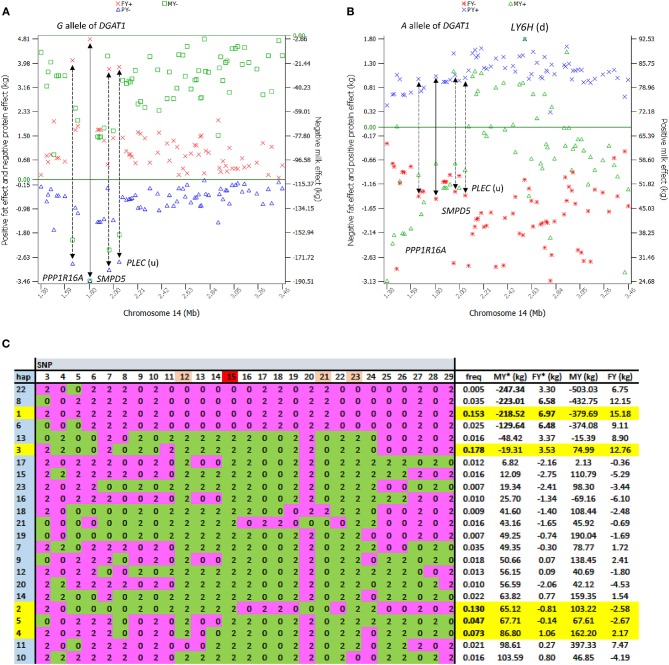
Extreme antagonistic pleiotropy effects of *DGAT1* and haplotypes of its surrounding regions. The antagonism of SNP effects between fat yield and milk and protein yields existed in the 2.08 Mb region of 1.43–2.24 Mb surrounding *DGAT1* but was most pronounced in or near four genes, *DGAT1, PPP1R16A, SMPD5*, and *PLEC*. **(A)** The *G* allele of *rs109421300* in *DGAT1* had the largest positive allele effect on fat yield and the largest negative effects on milk and protein yields. **(B)** The *A* allele of *rs109421300* in *DGAT1* had positive allele effects on milk and protein yields and negative effect on fat yield but the antagonism was not as strong as that of the *G* allele. **(C)** Haplotype analysis of 27 SNPs in the 1.18–2.19 Mb region among 398,845 Holstein cows showed that 23 haplotypes with high imputing confidence accounted for 88% of the haplotypes in this region. Haplotypes 3, 1, 2, 4, and 5 (highlighted in yellow) had the highest frequencies for a combined frequency of 0.58. The four haplotypes containing the “*G*” allele of *rs109421300* in *DGAT1* (“0” of SNP 15 in pink) had little variation, with haplotypes 22, 8, and 6 not having the alleles of haplotype 1 at only one or two SNPs. Haplotype 1 had the highest fat yield and was used as the reference haplotype. The remaining 19 haplotypes containing the “*A*” allele of *rs109421300* had variations at all 26 SNPs surrounding *DGAT1* and had different levels of milk and fat yields. Pink color: alleles in haplotype 1. Green color: alleles not in haplotype 1. MY = milk yield as phenotypic deviation from fixed non-genetic effects such as herd, year and season. FY = fat yield as phenotypic deviation from fixed non-genetic effects such as herd, year, and season. MY* = MY−2(milk PTA), and FY* = FY−2(fat PTA), where PTA = predicted transmitting ability from routine genetic evaluation. The MY*, FY*, MY, and FY values shown in the last four columns further subtracted the averages of haplotypes 24-12904. Haplotype imputing used FINDHAP with option to identify high confidence and low confidence haplotypes (VanRaden and Sun, 2014).

The 1.19–7.98 Mb region of Chr14 had two unique features: the extremely significant effects of *rs109421300* in *DGAT1* at 1,801,116 bp, which is 1,153 bp upstream of the *K232A* causal mutation (Grisart et al., 2004), and the large cluster of highly significant effects (Figure 2A). In our SNP data, *rs109421300* was the only SNP located in *DGAT1*. This SNP had extreme antagonistic pleiotropy between fat yield and milk and protein yields among all SNPs (Table 1; Figure 3; Figure S5). This antagonism was in agreement with previous report about *DGAT1*'s antagonistic pleiotropy based on four candidate causal variants in 858 German Holstein bulls (Thaller et al., 2003) and causal alleles of *DGAT1* in 3028 U.S. Holstein bulls (da Silva et al., 2010), but this study showed the extreme antagonism of *rs109421300* among all SNPs for the yield traits. The *rs109421300* SNP has *A* and *G* alleles. The *G* allele was responsible for the extreme antagonistic pleiotropy between positive fat yield and negative milk and protein yields, i.e., #1 for the highest fat yield with allelic effect of 4.81 kg, and #1 for the lowest milk and protein yields with −190.51 kg for milk yield and −3.46 kg for protein yield (Figure 3A). The *A* allele had antagonistic pleiotropy between negative fat yield and positive milk and protein yields but this antagonistic pleiotropy was far less strong than the antagonistic pleiotropy of the *G* allele. The effect rankings of the *A* allele were #139 for milk yield with allelic effect of 57.60 kg, #332 for protein yield with allelic effect of 1.04 kg, and #46 for the lowest fat yield with allelic effect of −1.46 kg (Figure 3B). Therefore, the significance of *DGAT1* for the yield traits was due to the extremely positive and negative effects of the “*G*” allele: #1 for positive fat effect, and #1 for negative milk and protein effects. The antagonistic pleiotropy between fat and milk yield as well as between fat and protein yield was present for all 41 SNPs in the 2.08 Mb region of 1,379,063–3,464,083 bp, but became weaker as the SNP position became farther away from *DGAT1* (Figures 3A,B). Four SNPs in *DGAT1, PPP1R16A, SMPD*5, and *PLEC* (the SNP is upstream of the gene) within this 2.08 Mb region had the strongest antagonism between positive fat yield and negative milk and protein yields, and the antagonism of the other SNPs in this region were much weaker. The antagonism between fat and milk yields and between fat and protein yields had nearly identical patterns (Figure S5). The fact that all 41 SNPs in this 2.08 Mb region had the same antagonism as *DGAT1* indicated that some of the SNP effects could be due to the linkage with *DGAT1* and that haplotypes containing the “*G*” allele of *rs109421300* in *DGAT1* would have less variations than haplotypes containing the “A” alleles of *rs109421300*. We examined haplotypes of 27 SNPs in the 1,189,341–2,194,228 kb region around *DGAT1* among 398,845 Holstein cows. The results showed that 23 haplotypes with high imputing confidence and the highest frequencies accounted for 88% of the haplotypes in this region. Haplotypes with the “*G*” allele of *rs109421300* in *DGAT1* had variation at only one or two SNPs, and mostly had the highest fat yield and the lowest milk yield. In contrast, haplotypes containing the “*A*” allele of *rs109421300* in *DGAT1* had variations at all the remaining 26 SNPs, and had different levels of milk and fat yields (Figure 3C). The lack of haplotype variations for haplotypes containing the “*G*” allele likely contributed to the large number of significant effects around *DGAT1*. The issue of linked and independent effects around *DGAT1* is analyzed toward the end of this manuscript.

**Figure 4:**
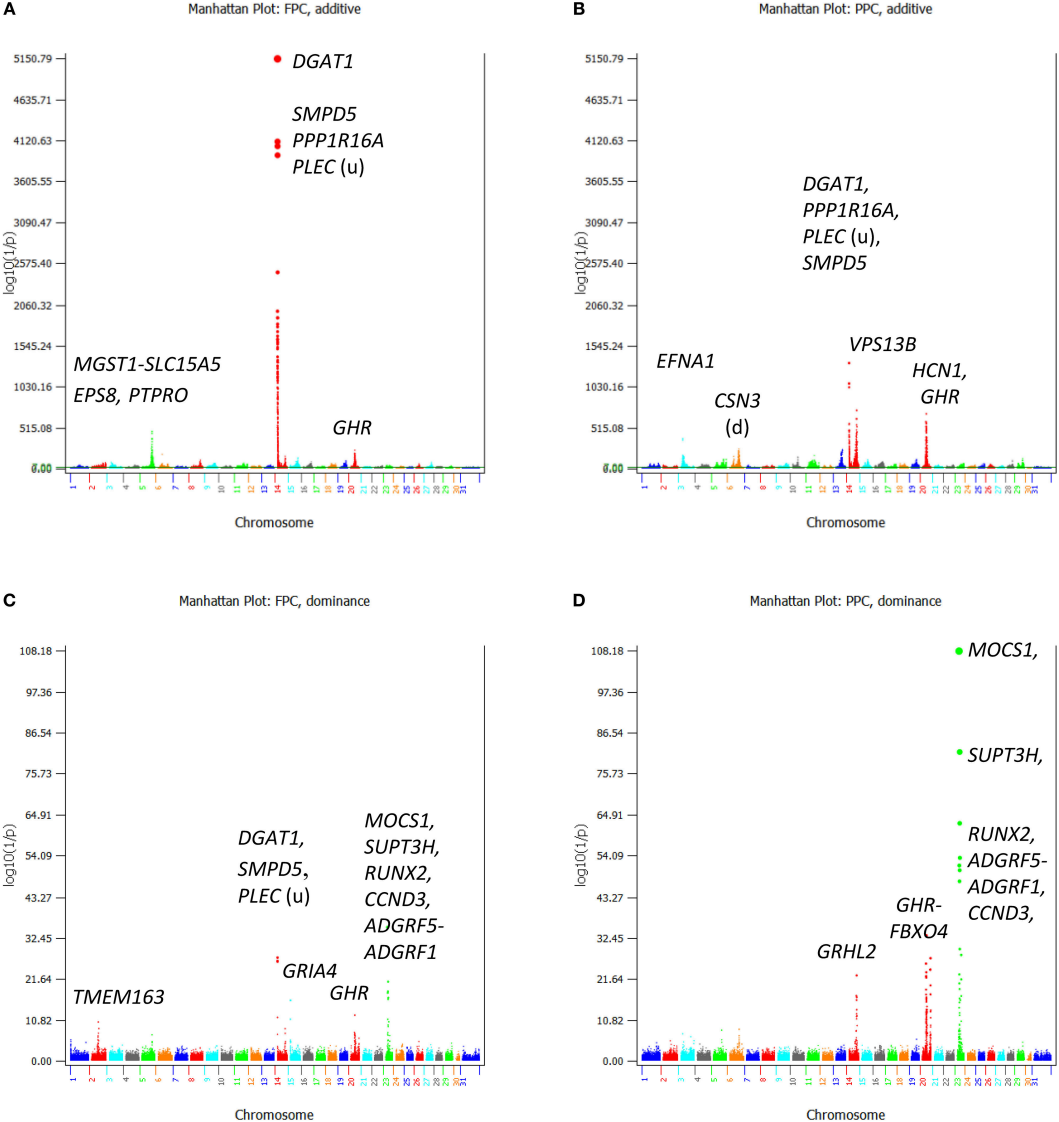
Statistical significance of additive and dominance SNP effects for fat and protein percentages. **(A)** Additive effects of fat percentage. **(B)** Additive effects of protein percentage. **(C)** Dominance effects of fat percentage. **(D)** Dominance effects of protein percentage. The horizontal line at log10(1/p) = 7 is the threshold for statistical significance of 5% genome-wide false positives with the Bonferroni correction.

Chr06 had two QTL regions with many highly significant SNP effects (Figures 4A-B), the 10.37 Mb region at 83.37–93.94 Mb and the 37.63–38.41 Mb region (Figure 2B). The 83.37–93.94 Mb region mostly affected milk and protein yields. The peak QTL effects were located at the 88.49–89.14 Mb region with 21 significant SNPs in and near four genes, *SLC4A4, GC, NPFFR2*, and *ADAMTS3*. This region also affected fertility traits and somatic cell score (as to be discussed). The 37.63–38.41 Mb region affected milk yield, and this region contained a previously reported causal gene for milk yield, *ABCG2* at 37.90 Mb (Cohen-Zinder et al., 2005).

Chr20 had significant SNP effects mostly on milk yield scattered over a large 19-Mb chromosome region at 23.86–42.21 Mb in the center of the chromosome with peak effects in the 30.03–36.67 Mb region (Figure 2C). The *GHR-PRLR* region had the largest concentration of significant effects on milk yield with peak QTL effects in *C6-PTGER4* downstream of *GHR*. This large QTL region was within the 28-Mb region of 21–49 Mb with the strongest evidence of selection signature by the analysis of extended haplotype homozygosity (Ma et al., 2019).

The large region of 83.69–102.28 Mb on Chr05 had a cluster of additive effects on fat yield with peak effects in or near *MGST1, SLC15A5, PLEKHA5*, and *ABCC9* (Figure 2D). In terms of statistical significance and the number of significant SNPs for fat yield, this region was second only to the 1.42–5.49 Mb region of Chr14 containing *DGAT1*. Two SNPs in *NDUFA*9 and *GALNT8* of Chr05 had highly significant additive SNP effects on milk and protein yields.

### Additive SNP Effects of Fat and Protein Percentages

For fat and protein percentages, the *rs109421300* SNP in *DGAT1* had the most significant effects (Figures 4A-B). This SNP had an unprecedented small *p*-values of 3.7(10^−10,871^) from BOLT-LMM and 6.2(10^−5,150^) from AGLS (Table 2). The significance of *rs109421300* SNP in *DGAT1* for fat percentage was intuitive, because *rs109421300* had largest positive effect for fat yield and the largest negative effect for milk yield, and the two *DGAT1* alleles had antagonistic pleiotropy between fat and milk yields (Figure 3). The extreme antagonistic pleiotropy between positive fat yield and negative milk yield of the “*G*” allele was the main contributor to *rs109421300's* extremely large effect on fat percentage, whereas the “*A*” allele's antagonism between positive milk effect and negative fat effect further added to the size of the average effect of gene substitution of fat percentage. Consequently, the average effect of gene substitution as the difference between these two allelic effects was the largest effect on fat percentage. The effect size of *rs109421300* in *DGAT1* for fat percentage was at least 2.75 times as large as the effect size of any SNP not on Chr14 based on the AGLS method, or at least 4.50 times as large based on BOLT-LMM.

**Table 2:** Significant additive effects of fat and protein percentages.

	**AGLS**							**BOLT-LMM**		
**SNP**	**Chr**	**Position (bp)**	**Candidate gene**	**Effect (α)**	***t*-value**	***p*-value**	**Rank**	**Effect (β)**	***p*-value**	**Rank**
**FAT PERCENTAGE**										
rs109421300	14	1801116	*DGAT1*	0.11	157.00	6.2E-5150^a^	1	0.18	3.7E-10872	1
rs109146371	14	1651311	*PPP1R16A*	0.10	140.00	1.7E-4109^a^	2	0.16	6.2E-8983	2
rs135549651	14	1967325	*SMPD5*	−0.10	139.00	3.6E-4051^a^	3	0.16	5.5E-8710	3
rs109350371	14	2054457	*PLEC* (u)	0.10	137.00	2.8E-3937^a^	4	0.15	7.2E-8321	4
rs109558046	14	2909929	*ARC-ADGRB1*	−0.07	108.00	4.8E-2463^a^	5	0.10	1.1E-5014	5
rs109558046	14	1489496	*ZNF34* (u)	−0.06	96.50	2.1E-1973^a^	6	0.10	7.2E-4363	6
rs110825637	5	93995487	*SLC15A5* (u)	0.03	46.00	5.9E-434^a^	85	0.05	4.5E-1099	76
rs133114040	5	94622206	*EPS8*	0.04	41.40	1.3E-372^a^	103	−0.04	7.2E-789	104
rs137735153	5	91136990	*PLEKHA5*	−0.02	35.40	2.3E-273	135	−0.04	1.1E-615	123
**PROTEIN PERCENTAGE**										
rs109421300	14	1801116	*DGAT1*	0.02	78.70	1.5E-1320	1	0.04	6.3E-3842	1
rs135549651	14	1967325	*SMPD5*	0.02	70.50	5.7E-1062	2	0.04	6.7E-3080	3
rs109146371	14	1651311	*PPP1R16A*	−0.02	70.50	5.7E-1062	3	0.04	1.2E-3231	2
rs109350371	14	2054457	*PLEC* (u)	0.02	68.90	5.4E-1015	4	0.04	3.6E-2911	4
rs109558046	14	67443766	*VPS13B*	−0.02	58.10	6.3E-725	5	−0.03	2.6E-1900	5
rs135228504	20	32394009	*LOC104975266* (d)	−0.02	56.30	1.4E-682	6	−0.03	3.8E-1848	6
rs109774038	20	293732441	*HCN1*	−0.02	52.00	1.8E-583	10	−0.03	2.5E-1536	13
rs41573457	20	30036600	*HCN1-GHR*	−0.02	51.70	4.3E-576	11	−0.03	8.2E-1471	15
rs132896414	20	32045791	*GHR*	−0.01	51.10	3.3E-563	12	−0.03	2.1E-1543	10

a This is the empirical p-value based on the observed t-value using the formula of $\text{lo}{\text{g}}_{10}\text{(1/p)=0}\text{.2416(}{\text{t}}^{\text{1}\text{.9713}}\text{)}$ (Figure S1), because the observed p-value was printed as “0” when the t-value is too large due to a limitation in the EPISNPmpi program. “u” indicates the SNP is upstream of the gene, and “d” indicates the SNP is downstream of the gene. “rank” is the rank of the statistical significance. “effect” is the average effect of gene substitution or the difference between allelic effects of “allele 1” and “allele 2.”

Contrary to the intuitive effect on fat percentage, the most significant effect of *rs109421300* in *DGAT1* for protein percentage was non-intuitive and could be misleading, because the “*G*” allele of *rs109421300* had the lowest milk and protein yields among all SNPs. Similar misleading results for protein percentage existed, including the SNP effects on protein percentage in *HCN1* and *GHR*. A SNP (*rs132896414*) in *GHR* had a significant effect for protein percentage (Table 2), but this SNP had low milk and protein yields. Therefore, the significance of this *GHR* SNP on protein percentage apparently was due to the very low milk yield, not due to high protein yield. Although SNPs with large effects for fat percentage and low fat and milk yields also existed, SNPs with the most significant and largest effects on fat percentage generally had the most significant and largest effects on fat yield as well. The SNP alleles in *PTPRO* and *EPS8* of Chr05 with large effects on protein percentage and low protein and milk yields had consistently large effects on fat yield and percentage, the 5 and 6th largest effects for fat yield, and the 9 and 10th largest effects for fat percentage. SNP effects with significant effects for both protein yield and percentage included those in or near *ADGRL3* and *TECRL* of Chr06, *PRLR* of Chr20, *GHRHR* of Chr04, and *VPS13B* of Chr14.

### Additive Effects of Fertility Traits and Somatic Cell Score

**Figure 5:**
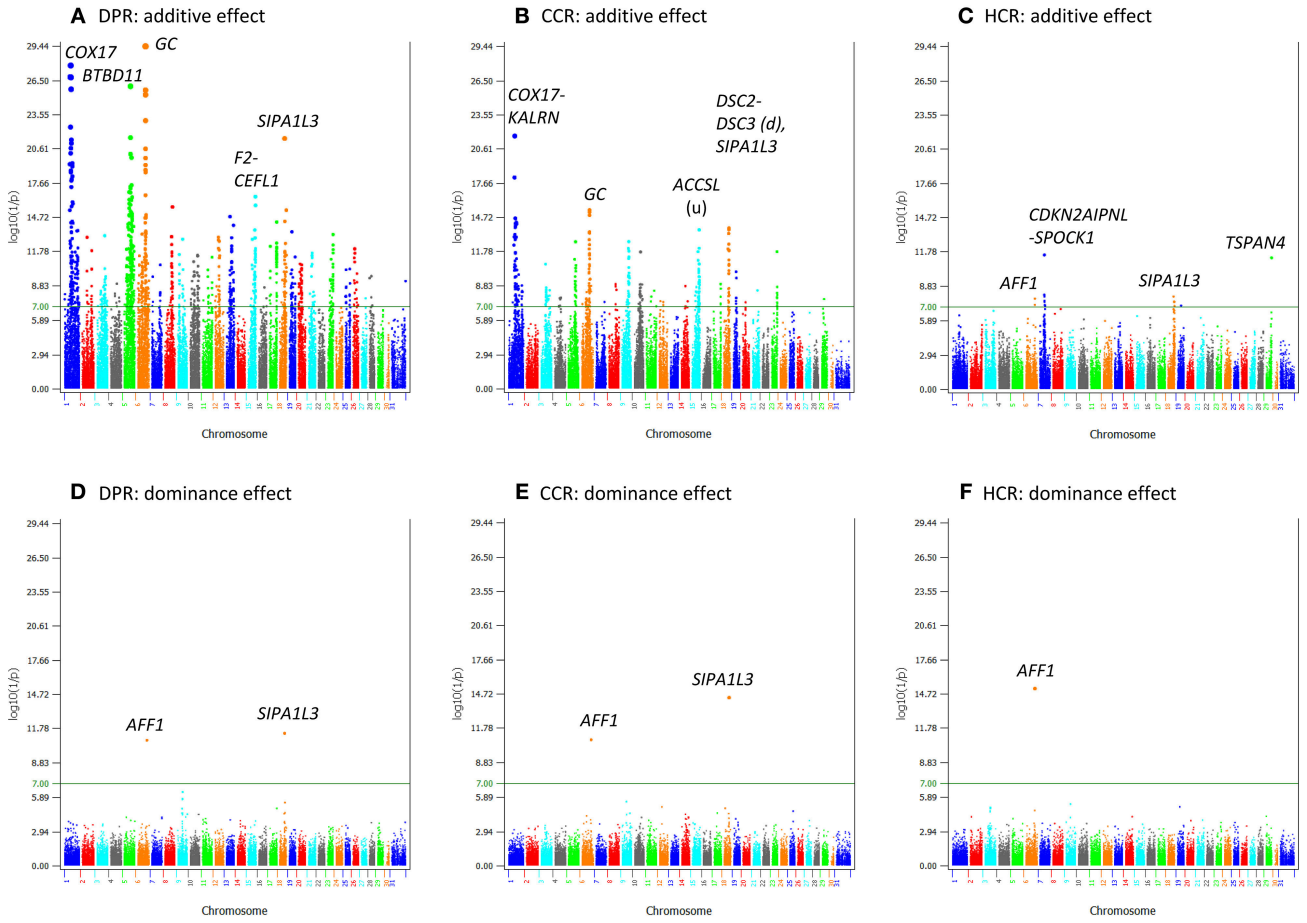
Statistical significance of additive and dominance SNP effects for fertility traits. **(A)** Additive effects of daughter pregnancy rate. **(B)** Additive effects of cow conception rate. **(C)** Additive effects of heifer conception rate. **(D)** Dominance effects of daughter pregnancy rate. **(E)** Dominance effects of cow conception rate. **(F)** Dominance effects of heifer conception rate. The horizontal line at log10(1/p) = 7 is the threshold for statistical significance of 5% genome-wide false positives with the Bonferroni correction.

Compared to yield traits, fertility traits had much smaller effects as indicated by the much smaller *t*-values. The largest *t*-value was 62.80 for milk yield and was 11.40 for daughter pregnancy rate, i.e., the largest milk effect was 62.80 times of its standard deviation whereas the largest effect for daughter pregnancy rate was only 11.40 times of its standard deviation. The number of additive effects exceeding the Bonferroni significance (*p* < 10^−7^) was 1,126 for daughter pregnancy rate, 360 for cow conception rate, and 15 for heifer conception rate (Table S5). Highly significant additive SNP effects included those in or near *GC* of Chr06, *COX17* of Chr01, and *SIPA1L3* of Chr18 for daughter pregnancy rate and cow conception rate; *BTBD11* of Chr05 for daughter pregnancy rate; a Chr15 region containing *ACCSL* for cow conception rate; and the *SLC25A48-IL9* region of Chr07 for heifer conception rate (Figures 5A–C; Table 3). A notable feature of the significant SNP effects on fertility was the large negative allelic effects. SNPs in the *COX17-ILDR1-KALRN* region of Chr01 and *SIPA1L3* of Chr18 had the most negative allelic effects on daughter pregnancy rate. SNPs in or near the *COX17-ILDR1-KALRN* region, *IGF2BP2* and *ELAVL4* of Chr01, *SIPA1L3* of Chr18, *NASP* of Chr03, and *DPY19L1, NPSR1, IMMP2L, ELMO1* of Chr04 had the most negative effects on cow conception rate; and *AFF1* had the most negative effect on heifer conception rate (Figure S4).

**Table 3:** Significant additive effects of fertility traits.

	**AGLS**							**BOLT-LMM**		
**SNP**	**Chr**	**Position (bp)**	**Candidate gene**	**Effect (α)**	***t*-value**	***p*-value**	**Rank**	**Effect (β)**	***p*-value**	**Rank**
**DAUGHTER PREGNANCY RATE (DPR)**										
rs133300430	6	88724389	*GC*	1.07	11.41	3.6E-30	1	1.05	6.5E-24	20
rs110254685	1	65025357	*COX17*	1.76	11.08	1.6E-28	2	−2.24	1.3E-36	1
rs108944043	1	60231667	*ZBTB20*	−1.43	10.87	1.6E-27	3	−1.64	4.5E-29	7
rs43244044	1	67115284	*ILDR1*	−1.37	10.87	1.7E-27	4	−1.63	8.9E-31	5
rs109894310	5	70997483	*BTBD11*	0.96	10.70	1.0E-26	5	0.82	1.6E-16	51
rs110966376	1	69574228	*KALRN*	1.38	10.65	1.8E-26	6	−1.76	5.0E-34	2
rs109901151	6	88494442	*SLC4A4*	−0.95	10.63	2.1E-26	7	−1.14	2.9E-30	6
rs109218398	5	71144630	*BTBD11*	0.97	9.71	2.7E-22	11	0.89	1.6E-16	57
rs110543856	18	48150900	*SIPA1L3*	1.60	9.70	3.1E-22	12	−2.00	1.4E-27	10
rs41572869	5	82738732	*PPFIBP1*	−0.85	9.30	1.5E-20	19	−0.90	3.9E-19	36
rs29009709	1	82032365	*IGF2BP2*	−1.21	9.18	4.3E-20	21	−1.55	2.7E-26	13
rs136965551	1	71581159	*UBXN7*	0.93	8.80	1.4E-18	31	−1.39	1.3E-32	3
rs41579094	1	71602911	*UBXN7* (d)	0.91	8.66	4.9E-18	34	−1.37	7.2E-32	4
**COW CONCEPTION RATE (CCR)**										
rs110254685	1	65025357	*COX17*	2.04	9.75	1.8E-22	1	−2.37	2.7E-24	1
rs109155375	1	62968592	–	1.68	8.88	6.8E-19	2	−1.10	2.5E-17	16
rs133300430	6	88724389	*GC*	1.01	8.13	4.5E-16	3	1.21	5.7E-19	7
rs109447734	6	88887995	*GC* (d)	−0.95	8.08	6.8E-16	4	−1.13	3.4E-18	11
rs110527224	6	88592295	*GC* (u)	−0.96	8.00	1.2E-15	32	−1.16	3.8E-18	13
rs109447734	1	69435214	*KALRN*	1.06	7.93	2.3E-15	6	−1.12	5.4E-14	35
rs110693378	1	72844496	*ACAP2*	−0.98	7.92	2.3E-15	7	−1.08	1.1E-15	21
rs29009709	1	82032365	*IGF2BP2*	−1.36	7.83	5.1E-15	8	−1.68	2.8E-18	10
rs136965551	1	71581159	*UBXN7*	1.08	7.80	6.3E-15	9	−1.49	2.5E-22	2
rs42341093	1	82007029	*IGF2BP2*	−1.36	7.78	7.1E-15	10	−1.67	2.8E-18	12
rs41616008	1	82147822	*IGF2BP2*	1.35	7.78	7.1E-15	11	−1.68	2.4E-18	9
rs110966376	1	69574228	*KALRN*	1.32	7.70	1.4E-14	13	−1.82	1.7E-21	4
rs110543856	18	48150900	*SIPA1L3*	1.64	7.54	4.7E-14	20	−1.13	1.4E-17	15
rs43244044	1	67115284	*ILDR1*	−1.26	7.51	5.8E-14	21	−1.71	5.6E-20	5
rs41579094	1	71602911	*UBXN7* (d)	0.91	8.66	4.9E-18	34	−1.37	5.3E-22	3
rs109960856	1	72080335	–	1.11	7.04	1.9E-12	41	−1.56	4.5E-19	6
**HEIFER CONCEPTION RATE (HCR)**										
rs42599672	7	48908617	*SLC25A48-IL9*	0.66	6.97	3.1E-12	1	−0.72	2.4E-12	2
rs42195584	29	50586068	*TSPAN4*	0.70	6.89	5.4E-12	2	0.66	2.9E-09	8
rs42598500	7	48881790	*SLC25A48*	−0.57	5.70	1.2E-08	4	0.65	3.3E-09	9
rs137244569	18	54520875	*NPAS1*	0.59	5.70	1.2E-08	5	0.46	4.5E-05	278
rs43480825	6	103774451	*AFF1*	0.89	5.63	1.8E-08	6	−1.61	1.4E-20	1
rs43480805	6	103752356	*AFF1*	−0.51	5.42	6.1E-08	11	0.67	2.3E-11	3
rs109889673	7	50508931	*SPOCK1*	0.52	5.52	3.4E-08	9	−0.65	2.4E-10	4
rs109574014	7	58685318	–	0.55	5.37	8.1E-08	15	0.69	4.9E-10	5
rs41911772	19	34773688	*B9D1* (d)	0.39	3.97	7.1E-05	283	−0.64	1.6E-09	6

“*Effect” is the average effect of gene substitution or the difference between allelic effects of “allele 1” and “allele 2.” “rank” is the rank of the statistical significance*.

**Figure 6:**
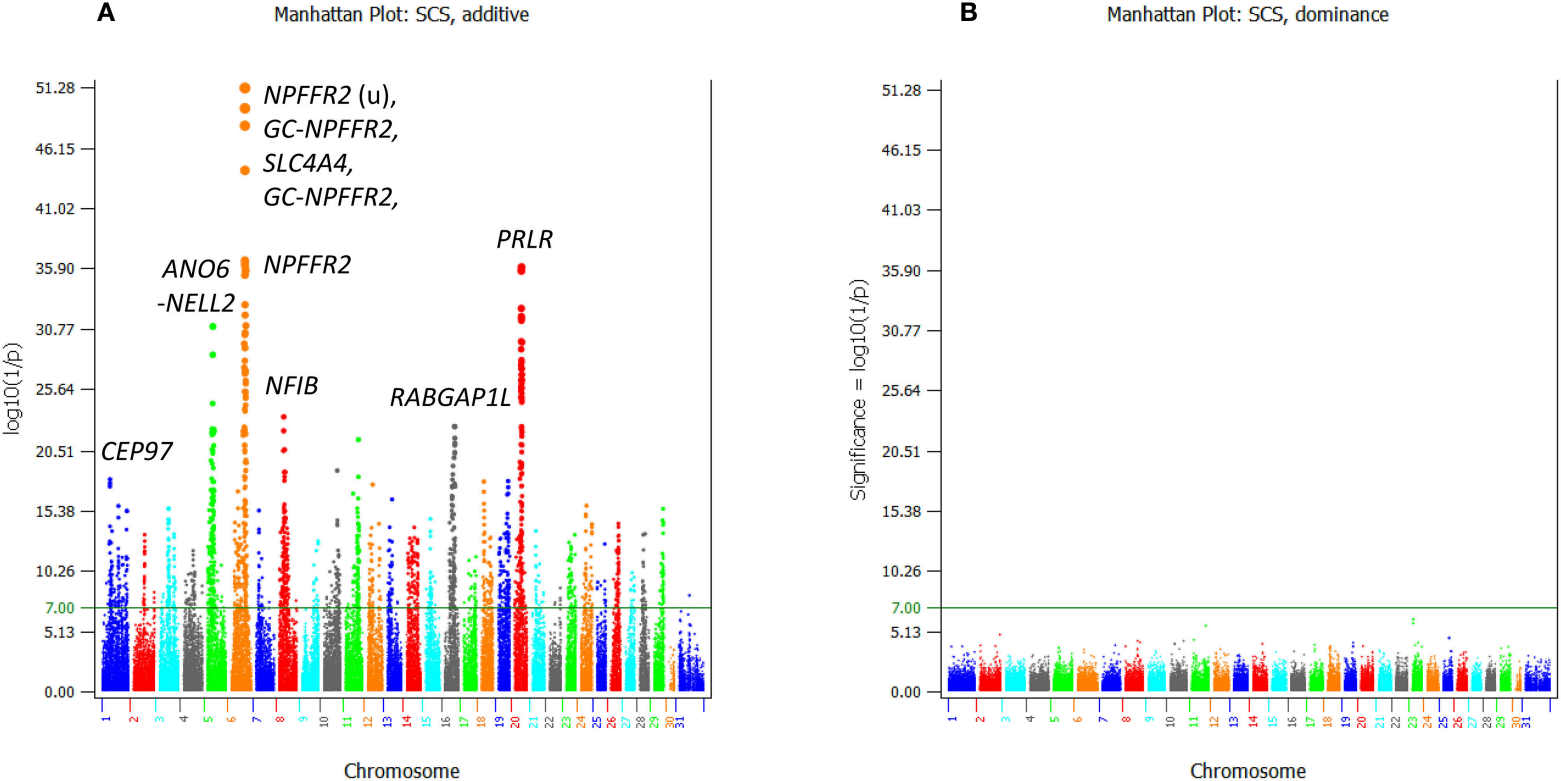
Statistical significance of additive and dominance SNP effects for somatic cell score. **(A)** Additive effects. **(B)** Dominance effects. The horizontal line at log10(1/p) = 7 is the threshold for statistical significance of 5% genome-wide false positives with the Bonferroni correction.

For somatic cell score, 2,348 additive effects (Table S5; Figure 6A) and no dominance effects exceeded the Bonferroni statistical significance (Figure 6B). SNPs in the *SLC4A4*-*GC*-*NPFFR2*-*ADAMTS3* region of Chr06 had the most significant additive SNP effects, followed by a SNP in *PRLR* of Chr20 (Table 4). SNPs in or near *CEP97, IMPG2*, and *ABI3BP* genes of Chr01 had the lowest allelic effects whereas SNPs in *ADAMTS3* of Chr06 and *PAPPA2* of chr16 had the highest allelic effects for somatic cell score (Table S5). However, the sizes of positive effects were not as large as the negative effects. Although the SNP in *CEP97* had the lowest somatic cell score, its ranking for statistical significance was only #141 because of the low allele frequency of 0.06 for the negative allele.

**Table 4:** Significant additive effects of somatic cell score.

	**AGLS**							**BOLT-LMM**		
**SNP**	**Chr**	**Position (bp)**	**Candidate gene**	**Effect (α)**	***t*-value**	***p*-value**	**Rank**	**Effect (β)**	***p*-value**	**Rank**
rs137147462	6	88887995	*GC* (d)	0.05	15.18	5.21E-52	1	0.08	2.7E-87	1
rs109452259	6	88800322	*GC* (d)	−0.05	14.91	2.80E-50	2	0.07	5.6E-73	3
rs110527224	6	88592295	*GC* (u)	0.05	14.68	8.45E-49	3	0.08	1.8E-76	2
rs109901151	6	88494442	*SLC4A4*	0.04	12.77	2.48E-37	5	0.07	1.2E-63	5
rs137844449	6	89050323	*NPFFR2*	0.05	12.69	7.30E-37	7	−0.06	2.0E-46	17
rs110243640	20	39017985	*PRLR*	0.04	12.67	9.40E-37	8	−0.06	2.1E-41	25
rs41588974	6	93157343	*SHROOM3*	0.05	12.62	1.78E-36	9	0.08	1.8E-64	4
rs41569309	20	37939597	*RNABP3L* (u)	−0.04	12.61	1.89E-36	10	0.06	9.2E-42	23
rs43671733	1	46436110	*CEP97*	0.06	8.83	1.04E-18	141	−0.07	2.2E-15	728

“*Effect” is the average effect of gene substitution or the difference between allelic effects of “allele 1” and “allele 2.” “u” indicates the SNP is upstream of the gene, and “d” indicates the SNP is downstream of the gene. “rank” is the rank of the statistical significance*.

### Dominance SNP Effects

Dominance effects were detected for all five milk production traits, but the statistical significance was much less than that of additive effects and the number of SNPs with dominance effects was much smaller than that of additive effects (Figures 1D–F; Figures 4C,D). The number of dominance effects exceeding the Bonferroni significance was 157, 81, 118, 25, and 117 for milk yield, fat yield, protein yield, fat percentage, and protein percentage, respectively (Table S6; Figure 6A). The smallest *p*-value for dominance effect was 4.8(10^−132^) for protein yield from AGLS (dominance effects unavailable from BOLT-LMM), compared to the smallest *p*-value of 6.2(10^−5, 150^) for additive effects of fat percentage. The much smaller number of significant dominance effects indicated that additive effects were the primary effects underlying the nine dairy traits.

Chr05 had the most significant and the largest number of dominance SNP effects on milk, fat and protein yields (Figures 1D–F; Table 5 and Table S6). SNPs in *AAAS* and *ATF7* at the downstream end and *PLXNC1* at the upstream end of the 24.11–27.11 Mb region had the most significant dominance effects on the yield traits. A second Chr05 region at 106.89–108.9 Mb in or near *PRMT8, TSPAN11, SLC6A13*, and *ERC1* also had significant dominance SNP effects on the yield traitss (Figures 1D–F). For fat and protein percentages, SNPs in or near two Chr23 genes (*MOCS1* and *SUPT3H*) and three Chr14 genes (*SMPD5, DGAT1*, and *PLEC*) had the most significant dominance effects for fat percentage, and five Chr23 SNPs in *MOCS1, SUPT3H*, RUNX2, *ADGRF5-ADGRF1*, and *CCND3* had the most significant dominance effects for protein percentage (Figures 4C,D; Table 6). Overdominance was the main effect type of the most significant dominance effects. For the example of milk yield, the top 34 dominance SNP effects were all positive overdominance effects because the heterozygous genotypic value of each SNP was higher than either homozygous genotypic values of the SNP. For fat and protein percentages, the significant dominance SNP effects in or near *MOCS1, SUPT3H, CCND3*, and *RUNX2*0 of Chr23 were negative overdominance effects, i.e., the heterozygous genotypic value of each SNP was lower than either homozygous genotypic values of the SNP. These SNPs also had significant positive overdominance effects on milk yield, consistent with their negative dominance effects on protein percentage.

**Table 5:** Significant dominance effects of yield traits.

**Trait**	**SNP**	**Chr**	**Position (bp)**	**Candidate gene**	**Effect (δ, kg)**	***t*-value**	***p*-value**	**Rank**
MY	rs11055819	5	26876852	*AAAS*	350.19	24.50	3.5E-115	1
MY	rs109438971	5	27130695	*EIF4B* (d)	347.20	22.63	2.64E-113	2
MY	rs109675908	5	26661043	*ATF7*	222.09	17.54	7.46E-69	3
MY	rs110384471	5	24111835	*PLXNC1*	254.33	17.14	7.97E-66	4
FY	rs11055819	5	26876852	*AAAS*	12.11	21.00	1.14E-97	1
FY	rs109438971	5	27130695	*EIF4B* (d)	12.02	20.80	1.98E-96	2
FY	rs109675908	5	26661043	*ATF7*	7.76	16.30	7.66E-60	3
FY	rs110384471	5	24111835	*PLXNC1*	8.981	16.10	1.30E-58	4
PY	rs11055819	5	26876852	*AAAS*	10.39	24.50	4.89E-132	1
PY	rs109438971	5	27130695	*EIF4B* (d)	10.39	24.40	3.11E-131	2
PY	rs109675908	5	26661043	*ATF7*	6.71	19.10	1.29E-81	3
PY	rs110730614	5	26561662	*ATF7* (u)	6.49	18.70	8.04E-78	4
PY	rs110384471	5	24111835	*PLXNC1*	7.67	18.60	3.29E-77	5

*MY, milk yield; FY, fat yield; PY, protein yield*.

**Table 6:** Significant dominance effects of percentage and fertility traits.

**Trait**	**SNP**	**Chr**	**Position (bp)**	**Candidate gene**	**Effect (δ)**	***t*-value**	***p*-value**	**Rank**
FPC	rs109266279	23	13846320	*MOCS1*	−0.04	12.50	7.20E-36	1
FPC	rs135549651	14	1967325	*SMPD5*	0.01	10.90	6.99E-28	2
FPC	rs109421300	14	1801116	*DGAT1*	0.01	10.80	5.24E-27	3
FPC	rs109350371	14	2054457	*PLEC* (u)	0.01	10.70	8.28E-27	4
FPC	rs110993492	23	18600456	*SUPT3H*	−0.02	9.52	1.71E-21	5
PPC	rs109266279	23	13846320	*MOCS1*	−0.02	22.20	6.67E-109	1
PPC	rs110993492	23	18600456	*SUPT3H*	−0.02	19.20	3.12E-82	2
PPC	rs43480825	23	18695002	*RUNX2*	−0.01	16.80	2.13E-63	3
PPC	rs43705624	23	20166517	*ADGRF5-ADGRF1*	−0.01	15.50	3.02E-54	4
PPC	rs43480825	23	15740337	*CCND3*	−0.01	15.20	3.17E-52	5
DPR	rs110543856	18	48150900	*SIPA1L3*	3.11	6.93	4.35E-12	1
DPR	rs43480825	6	103774451	*AFF1*	2.30	6.73	1.70E-11	2
CCR	rs110543856	18	48150900	*SIPA1L3*	4.78	7.86	3.94E-15	1
CCR	rs43480825	6	103774451	*AFF1*	3.03	6.73	1.66E-11	2
HCR	rs43480825	6	103774451	*AFF1*	3.12	8.08	6.43E-16	1

*FPC, fat percentage; PPC, protein percentage; DPR, daughter pregnancy rate; CCR, cow conception rate; HCR, heifer conception rate; “rank” is the rank of the statistical significance*.

The fertility traits had a small number of dominance effects exceeding the Bonferroni significance (*p* < 10^−7^), with only 2, 2, and 1 dominance effects for daughter pregnancy rate, cow conception rate, and heifer conception rate, respectively (Figures 5D–F; Table 6). The differences between additive and dominance effects in statistical significance were not as much as for production traits. The smallest *p*-values for additive effects were 10^−29.4^, 10^−21.7^, and 10^−11.5^ (Table 3) compared to 10^−11.4^, 10^−14.4^, and 10^−15.2^ for dominance effects of daughter pregnancy rate, cow conception rate, and heifer conception rate, respectively (Table 6). A SNP (*rs110543856*) in *SIPA1L3* of Chr18 and a SNP (rs43480825) in *AFF1* of chr06 were the only two SNPs with dominance effects exceeding the Bonferroni significance (*p* < 10^−7^) for daughter pregnancy rate, cow conception rate; and heifer conception rate (Figures 5D–F; Table 6). The dominance effect of *rs43480825* in *AFF1* of Chr06 was the most significant SNP effect for heifer conception rate among all additive and dominance effects for this trait [*p* = 6.43(10^−16^), Table 6].

### Types and Sizes of SNP Effects and Allelic Effects

**Figure 7:**
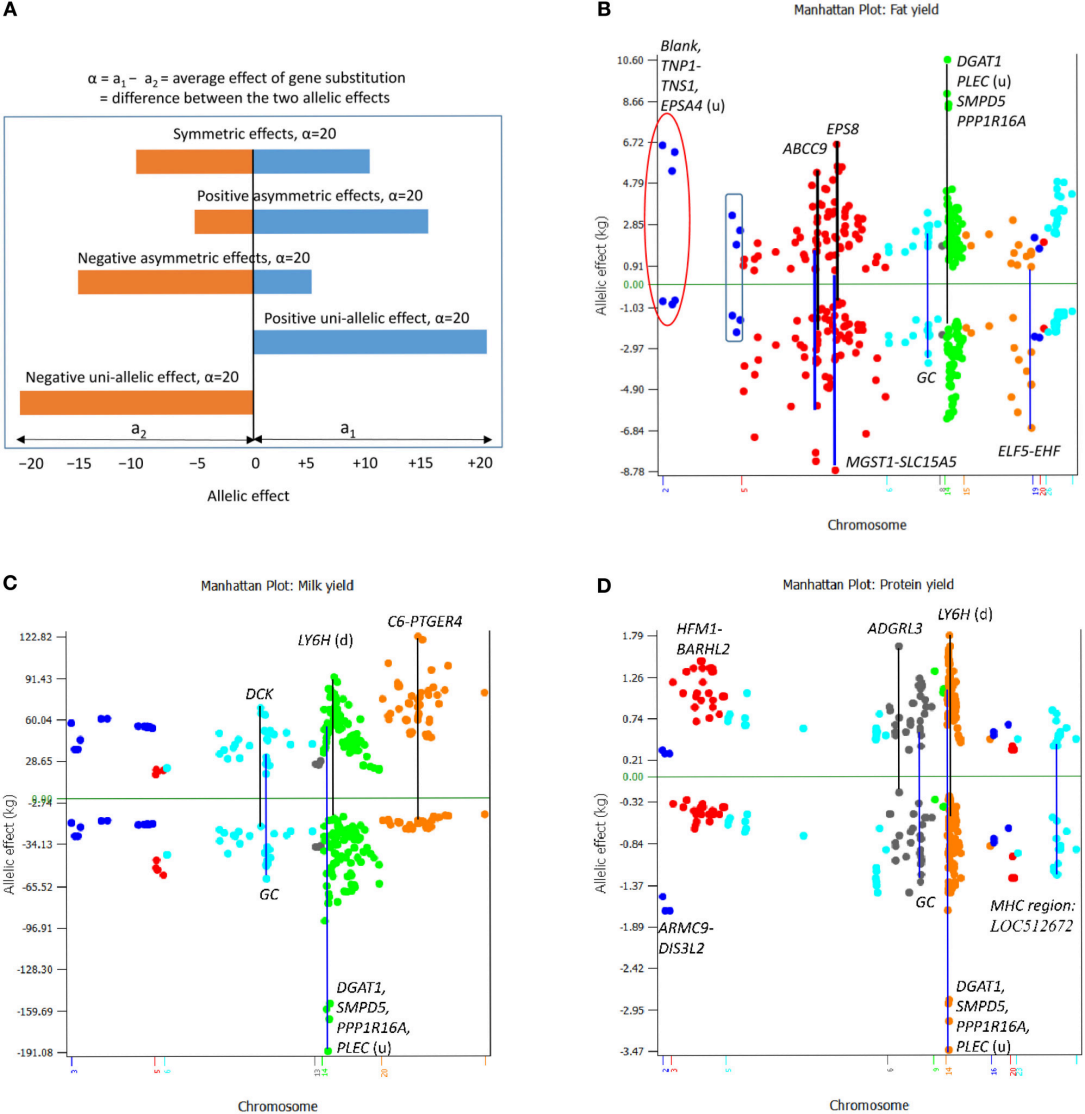
Conceptual types of allelic effects and observed SNP allelic effects among 200 most significant SNPs for each yield trait. **(A)** Conceptual effect types that may involve symmetric allelic effects, positive and negative asymmetric allelic effects, and positive and negative uni-allelic effects. **(B)** All types of conceptual allelic effects were observed for fat yield. *DGAT1* and three other genes near *DGAT1* had the largest positive asymmetric SNP effects, two SNPs in *ABCC9* had both positive and negative asymmetric effect, a SNP between *MGST1* and *SLC15A5* and a SNP between *ELF5* and *EHF* had negative uni-allelic effects, three SNPs of Chr02 (circled) had positive uni-allelic effects, and another three SNPs of Chr02 (boxed) had nearly symmetric effects. **(C)** *DGAT1* and three other genes near *DGAT1* had the largest negative asymmetric SNP effect and the *C6-PTGER4* region of Chr20 had the most positive asymmetric SNP effects for milk yield. **(D)** *DGAT1* and three other genes near *DGAT1* had the largest negative asymmetric SNP effects, and the *LY6H* downstream region and the *ARC-ADGRB1* region had the most positive asymmetric effects for protein yield.

The effect of a SNP measured by the average effect of gene substitution (or the difference between the two allelic means) of the SNP generally was associated with the statistical significance of the SNP, i.e., the larger the absolute SNP effect, the more significant the SNP effect. However, for similar SNP effect sizes, allelic effects of different SNPs may have sharply different interpretations. For the same SNP effect size, the allelic effects can be symmetric effects, where the two alleles have similar allelic effects in opposite directions; asymmetric effects, where one allele has a larger effect size than the other allele; or uni-allelic effect, where only one allele has effect while the other allele is a neutral allele and has no effect (Figure 7A). All these types of allelic effects were observed for the examples of 200 most significant SNPs per yield trait (Figures 7B–D).

For fat yield (Figure 7B), *rs109421300* in *DGAT1* of Chr14 had the largest positive asymmetric effect, whereas SNPs in *ABCC9* of Chr05 had both positive and negative asymmetric effects. The allelic effects of the three SNPs of Chr02 (red circle in Figure 7B), a SNP between *MGST1* and *SLC15A5* as well as a SNP in *EPS8* of Chr05 were close to having uni-allelic effects each with one large positive or negative allelic effect and one allelic effect close to the zero line. Three SNPs of Chr02 (boxed in Figure 7B) had relatively symmetric effects. For milk yield (Figure 7C), *rs109421300* in *DGAT1* had the most negative asymmetric effects, *rs41938455* in *C6* of Chr20 had the most positive asymmetric effects, and most of the SNP effects of Chr20 had positive asymmetric effects. For protein yield (Figure 7D), *rs109421300* in *DGAT1* had the most negative asymmetric effects, SNPs in the *HFM1-BARHL2* region of Chr03 had the most positive asymmetric effects, and SNPs in *ADGRL3* of Chr06 and the *ARMC9-DIS3L2* region of Chr02 had negative uni-allelic effects. The SNPs in the *SLC4A4-GC*-*NPFFR2*-*ADAMTS3* region of Chr06 collectively had the most symmetric effects for milk, fat, and protein yields (Figures 7B–D). Large allelic effects for fat and protein percentages, the three fertility traits, and somatic cell score are shown in Figure S4. Among the symmetric, asymmetric and uni-allelic effects, extreme asymmetric allelic effects such as the effect of *DGAT1* and uni-allelic effects such as the SNP upstream of *SLC15A5* would be more valuable than symmetric allelic effects that are not among the largest in either direction. These results of SNP and allelic effects along with the allelic analysis that revealed the extreme antagonistic pleiotropy of *DGAT1* showed that the analysis of allelic effects could yield valuable understanding of the SNP effects unavailable from statistical significance alone. These examples showed that the integrated analysis of statistical significance and effect size increased the understanding of the QTL effects.

Majority of the highly significant SNP effects also had large allelic effects. Such SNP effects included those in *DGAT1* for all five production traits, in *C6* for milk yield, in *ABCC9* for fat yield, in *COX17* for daughter pregnancy rate, and in *AFF1* for heifer conception rate. An example of inconsistency between statistical significance and the size of allelic effect was the effect of *rs43671733* in *CEP97* of Chr01 with the lowest somatic cell score and #141 ranking in statistical significance (Table 4). Reasons for large allelic effects not ranking high in statistical significance included asymmetric and uni-allelic effects that do not have large differences between the two allelic effects of the SNP, and extreme allele frequencies. The *t*-test of additive effects by the AGLS method accounts for variations associated with allele frequencies and none of the SNPs with rare alleles was among the most significant SNPs for any trait. Consequently, SNPs with extreme frequencies and large effects were not as significant as SNPs with similar allele effect sizes and medium allele frequencies. Such examples included *rs43480825* in *AFF1* with the largest effect size for heifer conception rate and allele frequency of 0.10 for its negative allele, and *rs43671733* in *CEP97* with the largest effect size for somatic cell score and allele frequency of 0.06 for its negative allele. The *AFF1* SNP was ranked #6 (Table 3) and the *CEP97* SNP was ranked #141 (Table 4), although both SNPs had the largest effect sizes of the traits. Therefore, requiring a MAF in the *t*-test by AGLS generally was unnecessary. However, the estimation of allelic effects or the average effect of gene substitution does not account for variations associated with allele frequencies, and many rare alleles had large effects. Some of those effects could be true given the existence of rare elite cows that should have their rare genetic variants but many of those rate allelic effects could be due to sampling and hence we use a MAF = 0.05 restriction for reporting allelic effects.

### Comparison With Previous GWAS Results

In comparing results in this study with those in other studies, it is important to note the differences between our study and the previous studies in sample size, number of SNPs, breeds, and phenotypic definitions for some traits. Here, we focus on the comparison with two recent GWAS in dairy cattle, a multibreed dairy GWAS using 632,003 SNP markers on 17,925 Holstein and Jersey cattle (Raven et al., 2014), and a Holstein GWAS using 17,300 bulls and over three million imputed SNPs (Weller et al., 2018). Given that the number of SNPs in this study was only a small fraction of those two studies, the confirmation between our study and those two studies was expected to be mostly the confirmation of the chromosome or gene regions rather than the confirmation of the exact variants.

*DGAT1*, which had been confirmed by numerous studies, was confirmed again by our study to contain the most significant effect for milk production, but our study further showed *DGAT1* had extreme antagonistic pleiotropy between fat yield and milk and protein yields at the genome-wide level and this antagonism extended to a 2.08 Mb region around *DGAT1*. In comparison with the multibreed dairy GWAS (Raven et al., 2014), our study confirmed SNP effects in *GC* of Chr06 for milk yield and in *GBA* of Chr03 for protein percentage, and confirmed several regions that were also significant in our study but not involving the same genes. These regions included *LOC782462* of Chr20 for milk yield, *MGST1* for fat yield and percentage, *CSN2* for protein yield, and *ESP8* for milk yield. The SNP effect in *LOC782462* was not highly significant in our study (#8447 in statistical significance), but was about 1.00 Mb downstream of *C6*, which was the most significant effect of Chr20 for milk yield in our study. *MGST1* had one SNP (*rs41595602*) in our study that was insignificant for fat yield (#36,311 in statistical significance). However, a SNP (*rs110825637*) only 0.42 Mb downstream of *MGST1* was the most significant effect of Chr05 for fat yield (Table 1), noting that the Chr05 region was the second most important region for fat yield identified by this study, after the *DGAT1* region. *CSN2* had no SNP in our study, but the region of 87.15–87.38 Mb with *CSN1S1, CSN2*, and *CSN3* had significant effects on protein yield with #205-#679 rankings in statistical significance [*p* = 7.92(10^−40^)−1.63(10^−25^)]. *ESP8* had a ranking of #456 (top 1% in statistical significance) for milk yield and was even more significant for fat yield (#81) in our study. Compared to the study using Holstein bulls (Weller et al., 2018), our study confirmed the *SLC4A4*–*GC-NPFFR2* region of Chr06 for somatic cell score, *MGST1* for fat percentage, and *GHR* for protein percentage. Our study partially confirmed the SNP at 103,202,217 bp of Chr06 for heifer conception rate because this SNP was 0.57 Mb upstream of the SNP at 103,774,451 bp in *AFF1* that had the most significant dominance effect by the AGLS method and the most significant additive effect by BOLT-LMM for heifer conception rate. Our study also found significant effects in the vicinity of previously reported causal variants, including *GHR* for milk production (Blott et al., 2003; Pausch et al., 2015) and *ABCG2* for milk yield and composition (Cohen-Zinder et al., 2005). We detected significant SNP effects in and around *GHR* for milk yield and protein percentage, and detected significant effects for milk yield in the *NAP1L5*-*HERC3* about 0.30 Mb upstream of *ABCG2* (Table 1; Figure 2B).

The SNP effects in the two recent GWAS (Bouwman et al., 2018; Weller et al., 2018) confirmed by this study were all in the four chromosomes of Figure 2. Except the Chr14 region containing *DGAT1* that was widely confirmed to have a large cluster of significant effects for milk production, the Chr05, Chr06, and Chr20 regions were all much larger regions and had many new SNP effects affecting more traits than in the previous reports. The Chr05 region at 83.69–102.28 Mb was 18.59 Mb in size, the Chr06 region of 83.37–93.94 Mb region was about 10.57 Mb in size, and the Chr20 region at 23.86–42.21Mb was 18.35-Mb in size. This large Chr20 QTL region was within the 28-Mb region of 21–49 Mb with the strongest evidence of selection signature by the analysis of extended haplotype homozygosity (Ma et al., 2019). In addition to the four chromosomes described in Figure 2, the additive SNP effects reported in this study involved all 29 bovine autosomes (Table S5), and identified additional regions with significant effects, including the 50.04–58.26 Mb of Chr03, the 105.45–106.36 Mb of chr05 and the 24.96–29.97 Mb of chr23 for protein yield; the 15.36–15.62 Mb of chr03 and the 64.00–71.84 Mb of Chr14 for protein percentage; the 60.23–72.08 Mb of Chr01 for daughter pregnancy rate and cow conception rate; and *AFF1* for heifer conception rate. Majority of the 2617 additive SNP effects involving 1472 SNPs (Table S5) and 494 dominance SNP effects involving 354 SNPs (Table S6) were new effects detected by this study.

### Factors Contributing to the Significant Effects

This large-scale GWAS had two unprecedented results: the large number of SNP effects exceeding the Bonferroni significance, and the extremely small *p*-values for the most significant effects. These unprecedented results could be due to several factors, the large sample size, the presence of many genetic variants underlying the phenotypes, accurate estimation and removal of non-genetic factors (such as herd, year, and season) from the phenotypic values, potential inflation of statistical significance of the AGLS and BOLT-LMM, and strong LD that have increased the number of significant effects.

The sample size of 294,079 cows was the largest for GWAS in any animal species. As sample size increases, the statistical power increases and the rate of false positives decreases for a given effect size, or the detectable effect size decreases for a given statistical power and a rate of false positives (Mao and Da, 2005). Therefore, the large sample size apparently was a contributing factor for the many significant SNP effects. The presence of many genetic variants underlying a phenotype is also a likely reason for the large number of SNP effects. The hypothesis of many genetic variants underlying a production is supported by the steady genetic progress in dairy genetic selection, which has more than doubled the milk yield from 5.3 tons in 1957 to 12.5 tons in 2015 (Figure S6). Although large sample size increases the ability to detect more variants than small samples, the number of SNP effects detected for the five production traits was surprisingly large, 58,207 additive effects from AGLS or 84,072 additive effects from BOLT-LMM, compared to the 4231 SNP effects for human height and body mass index (Yengo et al., 2018). The large number of genetic variants underlying the dairy production traits and the accurate estimation and removal of non-genetic factors (such as herd, year, and season) from the phenotypic values using large samples could have been the contributing factors to the large number of significant SNP effects. Potential inflation in statistical significance by both AGLS and BOLT-LMM could exist, although AGLS detected 46% fewer significant effects than BOLT-LMM. Genetic selection in Holstein cattle resulted in strong LD for many haplotypes that had high extended haplotype homozygosity for long chromosome distances (Ma et al., 2019). Such long haplotypes necessarily should have contributed to the number of significant effects through LD with causal effects.

**Figure 8:**
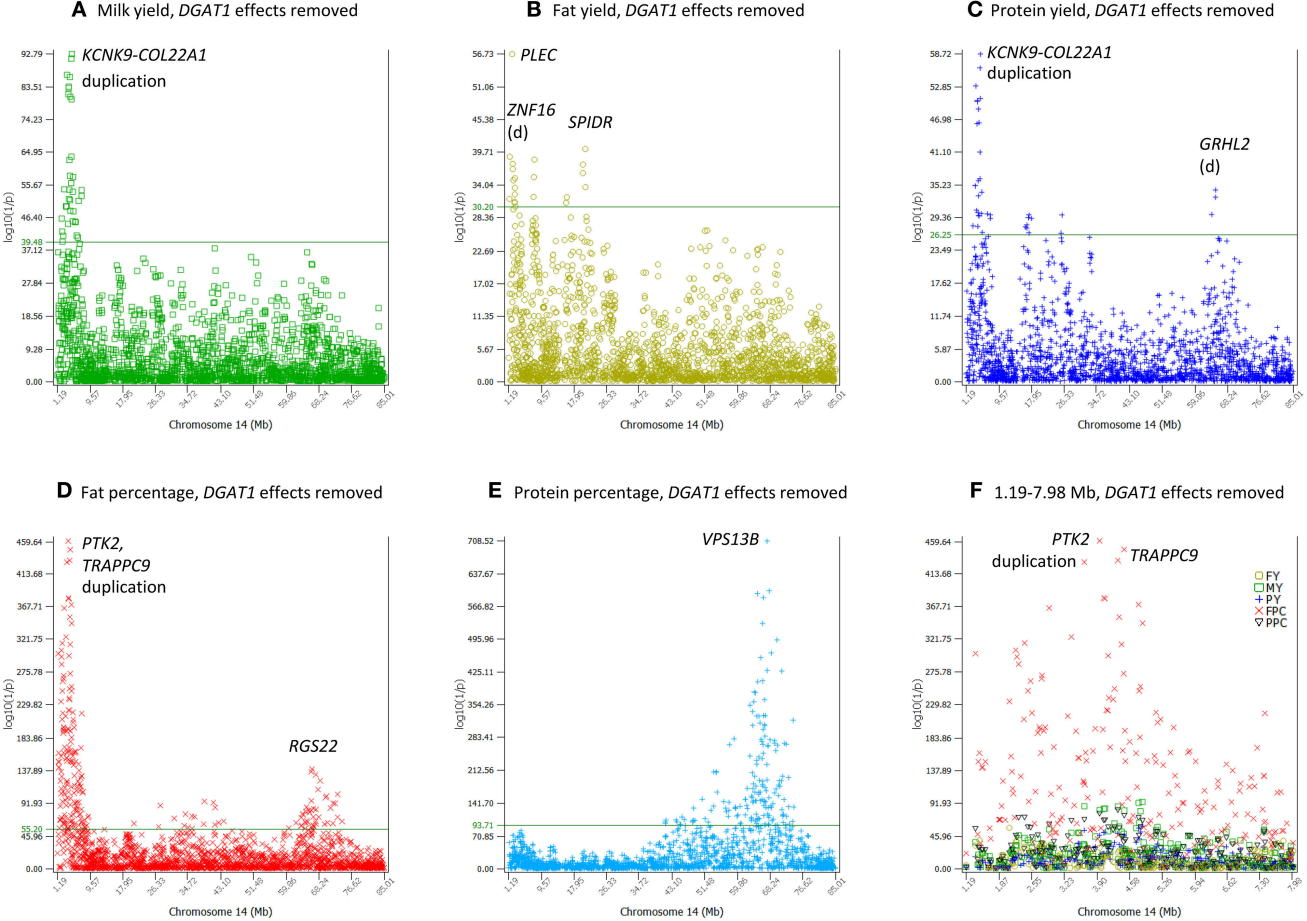
Chr14 SNP effects conditional on the removal of *DGAT1* effects from the phenotypic values for five production traits. The horizontal line on each graph is the threshold log_10_(1/p) value for the top 1% significant effects of each trait. The threshold value was 39.48 for milk yield, 30.20 for fat yield, 26.25 for protein yield, 55.20 for fat percentage, and 93.71 for protein percentage. The duplication is at Chr14:2932132-3866447. **(A–C)** Yield traits. The log_10_(1/p) values for the most significant effects decreased to 92.78, 56.73, and 58.72, compared to the log_10_(1/p) values of 820.00, 373.90, and 370.36 for milk, fat, and protein yields without removing the *DGAT1* effects, respectively. **(D)** Fat percentage was least affected by the removal of the *DGAT1* effects although the log_10_(1/p) for the most significant effect was reduced by about 10-folds, log_10_(1/p) = 459.64 for *rs41624797* in *PTK2*, compared to log_10_(1/p) = 5150.79 for *rs109421300* in *DGAT1* without removing the effects of *rs109421300*. **(E)** A large Chr14 region containing *VPS13B* became the most significant region with *DGAT1* effects removed, and became slightly more significant than without removing the *DGAT1* effects. **(F)** SNP effects for the five production traits in the 1.19–7.98 Mb region of Chr14. Fat percentage remained to have the most significant effects among all five traits of milk production with the removal of the *DGAT1* effects.

To assess the impact of LD on the number of significance effects and the significance levels, we analyzed the 1.19–7.98 Mb region of Chr14 containing *DGAT1* as an example. The analysis first estimated the genotypic effects of *rs109421300* in *DGAT1*, and then removed the estimated genotypic effects of *rs109421300* from the phenotypic values to produce the residual values for the five production traits. These residual values were used for association analysis using the AGLS method for all 2104 Chr14 SNPs. The results showed that the removal of the *DGAT1* effects represented by the *rs109421300* effects drastically reduced the number of significant effects and reduced the level of statistical significance for many SNPs, but also showed the existence of multiple genetic variants in the 1.19–7.98 Mb region affecting four of the five production traits (except protein percentage). The removal of the *DGAT1* effects eliminated about 63% (420 out of 666 SNPs) of the top 1% SNP effects for the five production traits (Table 7), indicating that 63% of the top 1% SNP effects in the 1.19–7.98 Mb region could have been due to LD with *DGAT1*. The level of statistical significance was drastically reduced for many but not all SNPs and some SNPs became more significant with the removal of the *DGAT1* effects (Table S7). For the three yield traits, the log_10_(1/p) values for the most significant effects decreased to 92.78, 56.73, and 58.72 (Figures 8A–C), compared to the log_10_(1/p) values of 820.00, 373.90, and 370.36 for milk, fat, and protein yields without removing the *DGAT1* effects, respectively. Fat percentage still had the largest number of top 1% effects among the five production traits, 154 effects with the removal of *DGAT1* effects and 188 effects without (Table 7; Figure 8D), indicating that many SNP effects on fat percentage independent of the *DGAT1* effects existed. Protein percentage was affected most by the removal of the *DGAT1* effects, i.e., all the previous 96 top 1% SNP effects were eliminated (Table 7). The most significant region associated with protein percentage was a large region around *VPS13B* about 65 Mb downstream of *DGAT1* and this region was unaffected by the removal of the *DGAT1* effects (Figure 8E). A SNP in *VPS13B* had the most significant effect on protein percentage among all SNPs after removing the *DGAT1* effects. The combined analysis of the SNP effects for the five production traits in the 1.19–7.98 Mb region of Chr14 showed that fat percentage remained to have the most significant effects among all five traits of milk production with the removal of the *DGAT1* effects (Figure 8F). The fact that 246 top 1% effects were still present after the removal of the *DGAT1* effects for the five production traits (Table 7) showed that multiple SNP effects independent of the *DGAT1* effects existed around *DGAT1*. This result along with the result of linked effects due to LD with *DGAT1* indicated that the large clusters of SNP effects in the 1.19–7.98 Mb region of Chr14 was a mixture of linked effects due to LD with *DGAT1* and the presence of multiple SNP effects independent of the *DGAT1* effects.

**Table 7:** Number of top 1% SNP effects in the 1.19–7.98 Mb region of Chr14 with and without removal of *DGAT1* effects from the phenotypic values for association analysis.

	**MY**	**FY**	**PY**	**FPC**	**PPC**	**Total**
*DGAT1* effects removed	38	15	39	154	0	246
*DGAT1* effects not removed	153	98	131	188	96	666

*MY, milk yield; FY, fat yield; PY, protein yield; FPC, fat percentage; PPC, protein percentage*.

## Conclusion

The results in this study provided large-sample confirmation of some previously reported SNP effects and chromosome regions associated with dairy traits, expanded some chromosome regions that contained reported SNP effects, detected a large number of new additive and dominance SNP effects and several new chromosome regions, and generated new understanding about the genetic mechanism of SNP effects affecting dairy traits. This study confirmed a small number of SNP effects from previous GWAS and confirmed several previously reported chromosome regions with SNP effects including a Chr14 region containing *DGAT1*, and the Chr05, Chr06, and Chr20 regions for milk production. For the Chr14 region, this study showed the extremely antagonism between fat yield and milk and protein yields of a SNP in *DGAT1* among all SNPs, identified SNPs with opposite effects to the *DGAT1* effects, and showed linkage disequilibrium with *DGAT1* contributed to a large number of significant effects around *DGAT1*. For the Chr05, Chr06, and Chr20 regions with previously reported QTL effects, this study identified those regions to be large regions of 10–19 Mb in size with QTL effects. The Chr20 region was the largest QTL regions affecting milk, the Chr06 regions affected all nine traits, and the Chr05 region was the second region with highly significant and the largest number of QTL effects on fat yield after the Chr14 region containing *DGAT1*. New QTL regions detected by this study included regions with the most significant or most negative SNP effects on fertility on Chr01, Chr03, Chr04, Chr06 and Chr18; and the Chr06, Chr20 and Chr05 regions for somatic cell score. Majority of the SNP effects for the nine dairy traits reported in this study were new effects including some in previously reported QTL regions. Additive effects were the main effects of all nine dairy traits. A relatively small number of new dominance effects mostly due to overdominance was detected for dairy production and fertility traits. The dominance effects generally were far less significant than additive effects with a few exceptions for the fertility traits. The integrated analysis of statistical significance with allelic effect size and direction provided new understanding of SNP effects, including extreme antagonistic pleiotropy, uni-allelic, asymmetric, and symmetric allelic effects.

## Author Contributions

LM, JJ, YD, PV, and JC conceived this study. JJ and LM prepared the data, and PV contributed to data preparation. JJ, LM, DP, and YD conducted the data analysis. JC provided thorough review and editing of the manuscript. YD, LM, and JC prepared the manuscript.

### Conflict of Interest Statement

The authors declare that the research was conducted in the absence of any commercial or financial relationships that could be construed as a potential conflict of interest.
