# Supplementary material for: A Large-Scale Genome-Wide Association Study in U.S. Holstein Cattle
Source: Front Genet. 2019 May 14;10:412. doi: 10.3389/fgene.2019.00412 (PMC6527781; doi:10.3389/fgene.2019.00412)
Supplement: Supplementary file 1 [file Data_Sheet_1.zip › Spplementary_material/Supplementary_material.pdf]

***Supplementary Material*:**

A large-scale genome-wide association study in U.S. Holstein cattle

FIGURE S1 | Empirical log(1/p) values as a power function of the observed t-values had a 100% correlation with the observed log(1/p) values from the GWAS.

FIGURE S2 | The two methods of BOLT-LMM and AGLS identified the same chromosome regions for nearly all highly significant additive SNP effects of nine dairy traits.

FIGURE S3 | Comparison of ranking in statistical significance and effect sizes between BOLT-LMM and AGLS.

FIGURE S4 | Comparison between statistical significance and allelic effects.

FIGURE S5 | The 2.08 Mb region of 1,379,063 to 3,464,083 bp of Chr14 containing *DGAT1* had nearly identical patterns of antagonism between fat yield and milk and protein yields.

FIGURE S6 | The 58 years of genetic selection during 1957-215 in US Holstein cattle more than doubled milk yield from 5.3 tons in 1957 to 12.5 tons in 2015 based on the annual milk yields of 87,729,358 Holstein cows.

TABLE S1 | Number of observations for nine dairy traits.

TABLE S2 | SNP chips for the GWAS population.

TABLE S3 | Birth years of the 294,079 Holstein cows for GWAS.

TABLE S4 | Number of SNP effects exceeding the statistical significance with the Bonferroni correction ($p<10^{-7}$) for nine dairy traits by AGLS and BOLT-LMM methods.

TABLE S5. List of significant additive SNP effects for nine dairy traits. (Excel)

TABLE S6. List of significant dominance SNP effects for eight dairy traits. (Excel)

TABLE S7 | Significant Chr14 additive effects after removal of *DGAT1* effects using AGLS method.

FIGURE S1 | Empirical log(1/p) values as a power function of the observed t-values had a 100% correlation with the observed log(1/p) values from the GWAS.

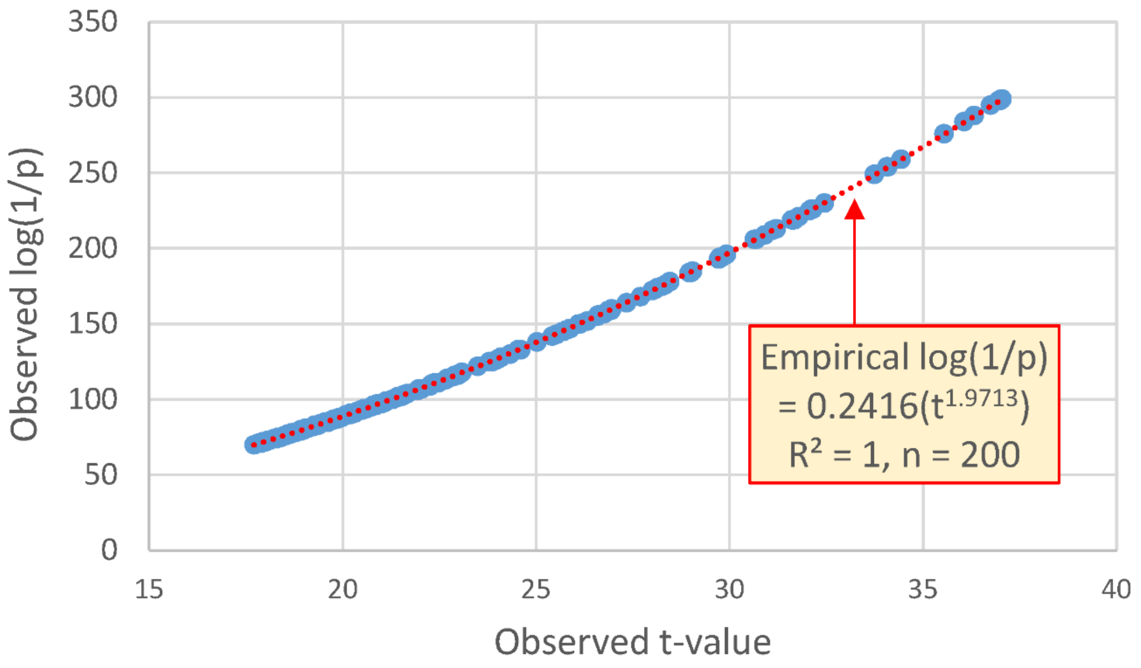

FIGURE S2 | The two methods of BOLT-LMM and AGLS identified the same chromosome regions for nearly all highly significant additive SNP effects of nine dairy traits. A gene name (e.g., *DGAT1*) indicates the gene at least had one significant SNP effect, and a'-' between two genes indicates the SNP effect was between these two genes. 'd' indicates the significant SNP effect is downstream of the gene. 'u' indicates the significant SNP effect is upstream of the gene. My = milk yield, FY = fat yield, PY = protein yield, FPC = fat percentage, PPC = protein percentage, SCS = somatic cell score, DPR = daughter pregnancy rate, CCR = cow conception rate, HCR = heifer conception rate.

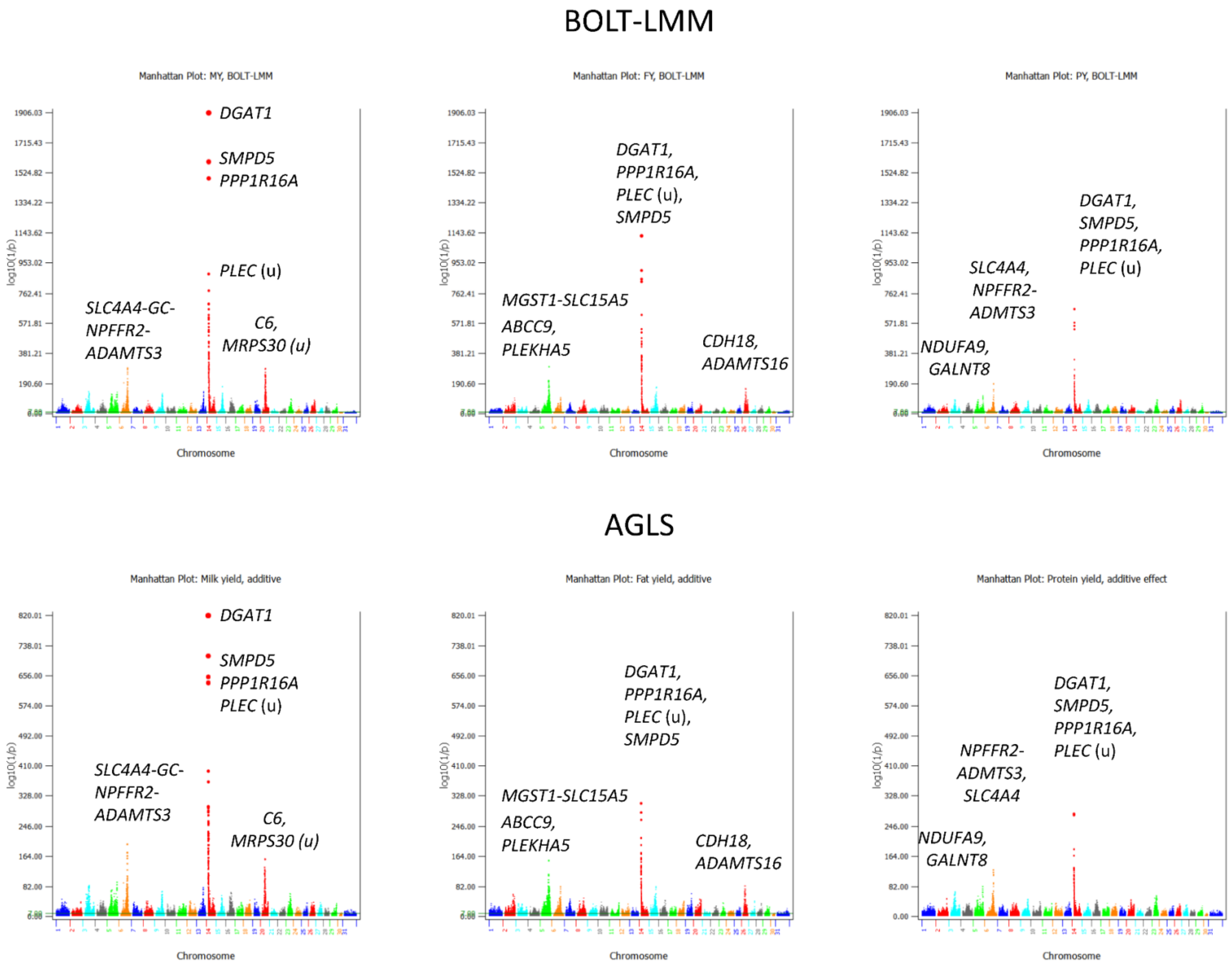

BOLT-LMM

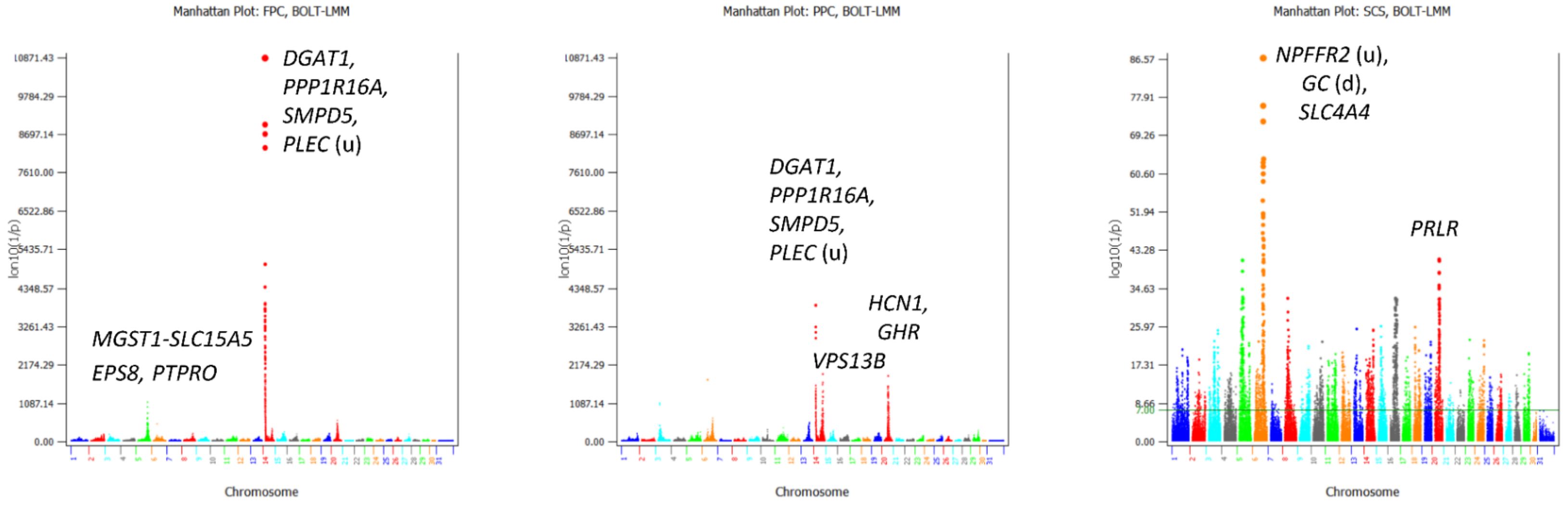

AGLS

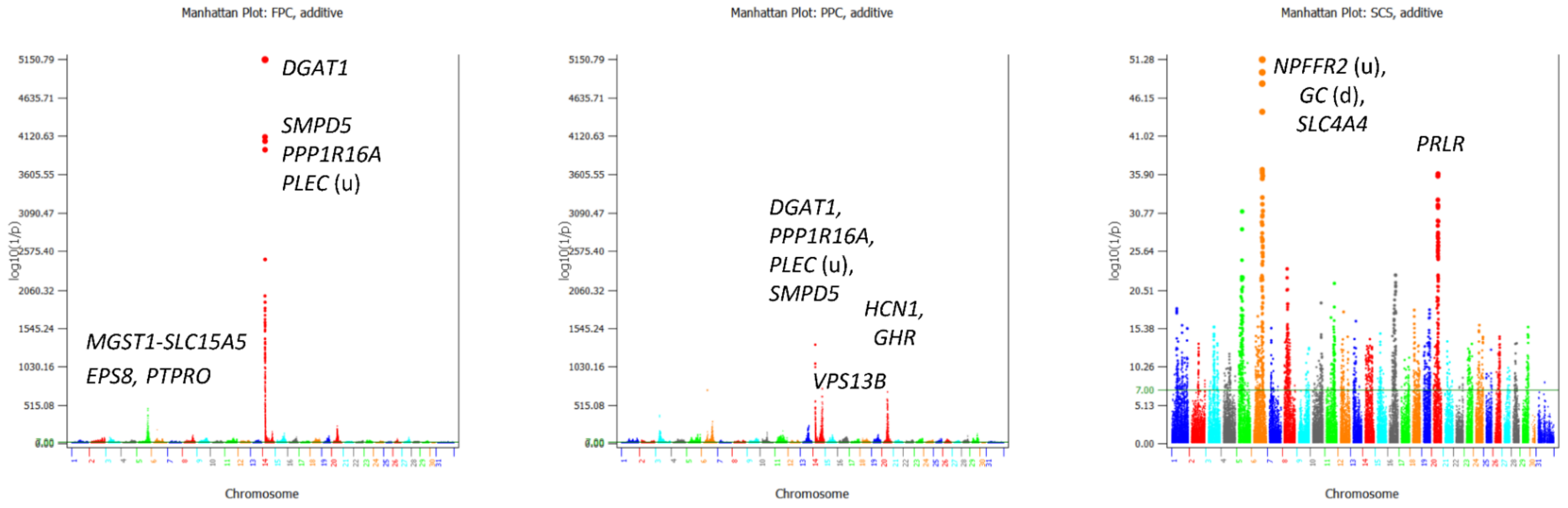

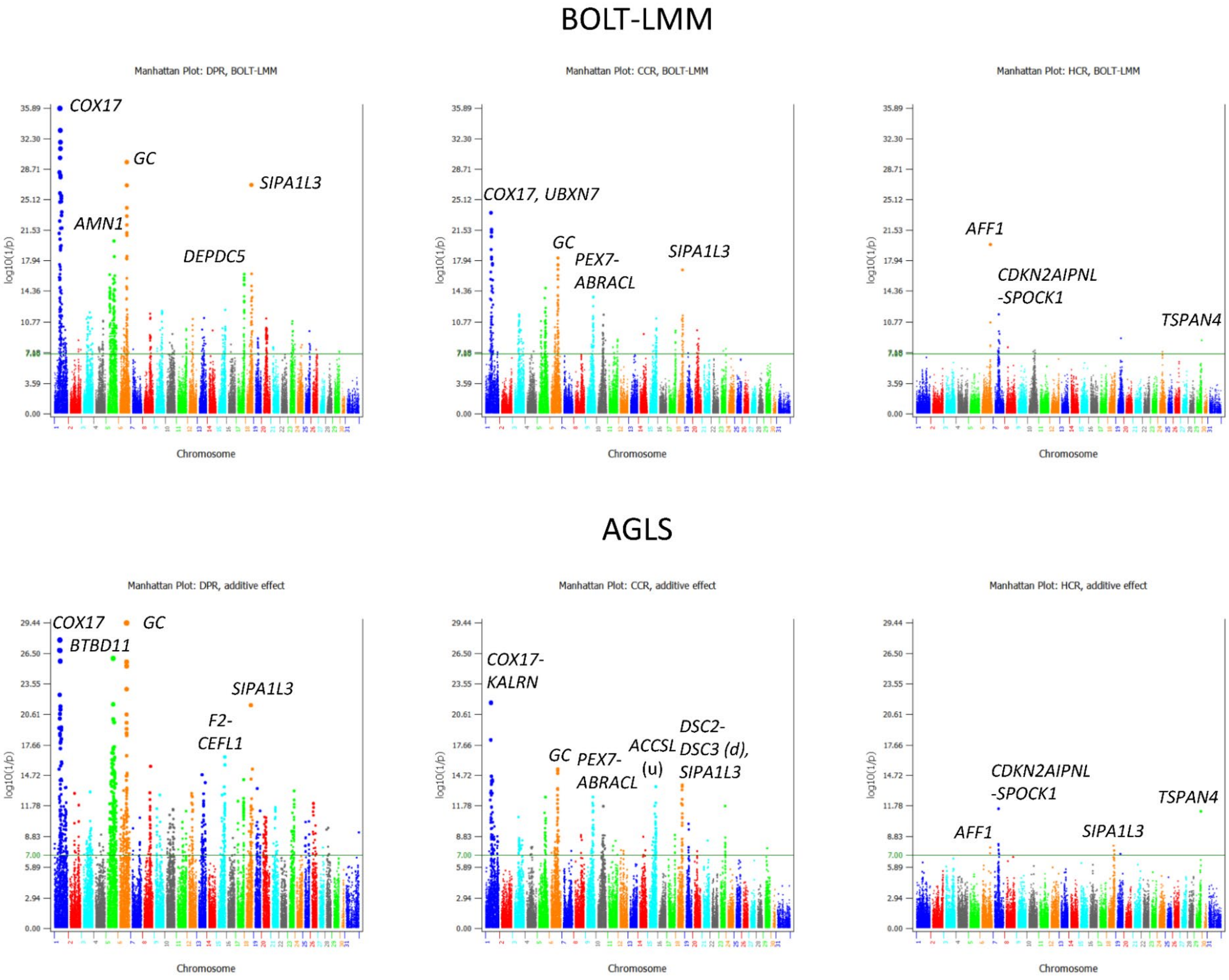
BOLT-LMM
Manhattan Plot: DPR, BOLT-LMM
COX17
GC
SIPA1L3
AMN1
DEPDC5
Manhattan Plot: CCR, BOLT-LMM
COX17, UBXN7
GC
PEX7-
ABRACL
SIPA1L3
Manhattan Plot: HCR, BOLT-LMM
AFF1
CDKN2AIPNL
-SPOCK1
TSPAN4
log10(1/p)
Chromosome
AGLS
Manhattan Plot: DPR, additive effect
COX17
GC
BTBD11
SIPA1L3
F2-
CEFL1
Manhattan Plot: CCR, additive effect
COX17-
KALRN
GC
PEX7-
ABRACL
ACCSL
(u)
DSC2-
DSC3 (d),
SIPA1L3
Manhattan Plot: HCR, additive effect
CDKN2AIPNL
-SPOCK1
TSPAN4
AFF1
SIPA1L3
log10(1/p)
Chromosome

FIGURE S3 | Comparison of ranking in statistical significance and effect sizes between BOLT-LMM and AGLS. The left column is the ranking of statistical significance and the right column is the effect sizes. AGLS_rank = rank of statistical significance by AGLS, BOLT_rank = rank of statistical significance by BOLT-LMM, AGLS_eff = effect size in absolute value of the gene substitution effect, BOLT_beta = effect size in absolute value of the regression coefficient from BOLT-LMM.

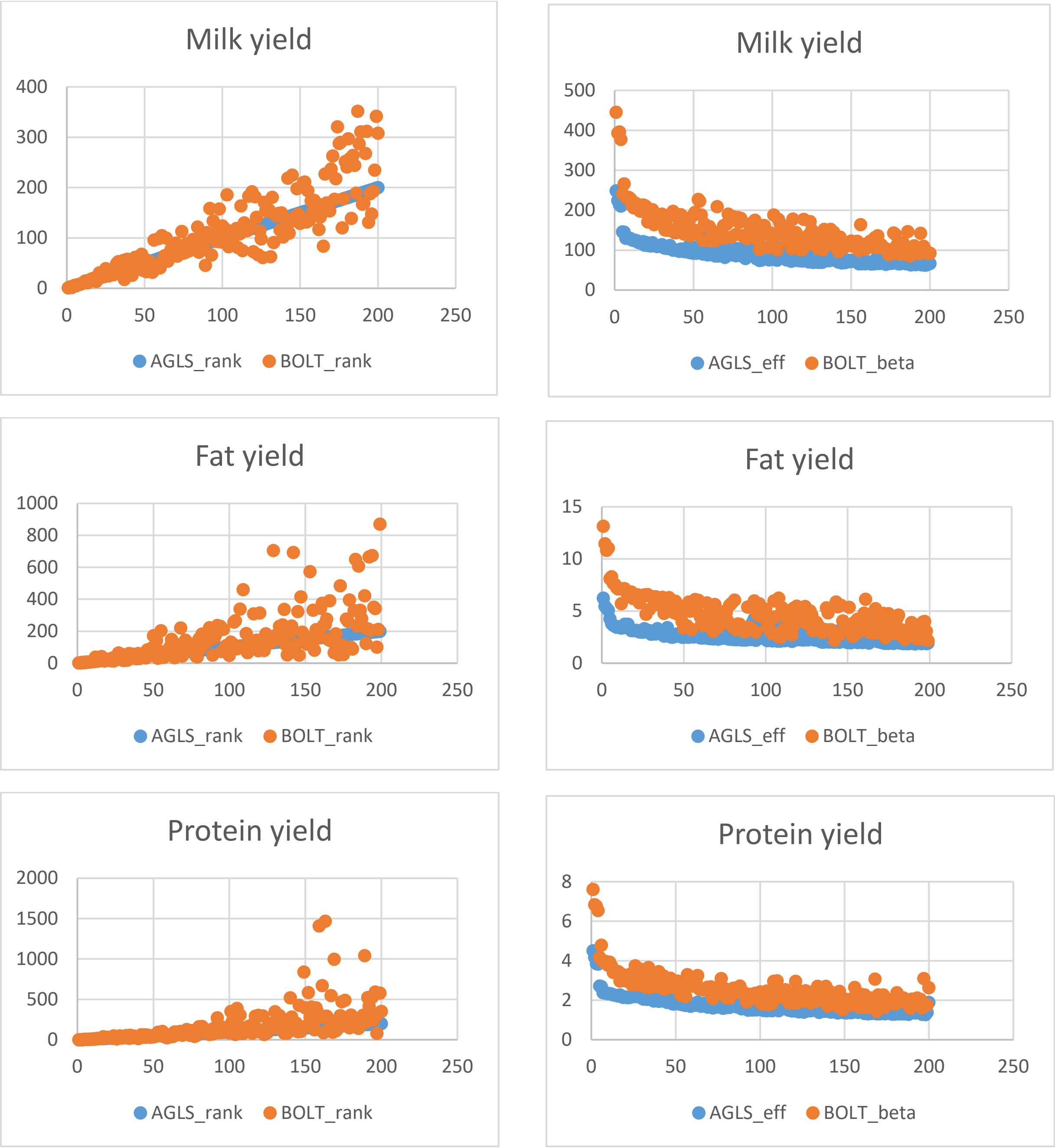

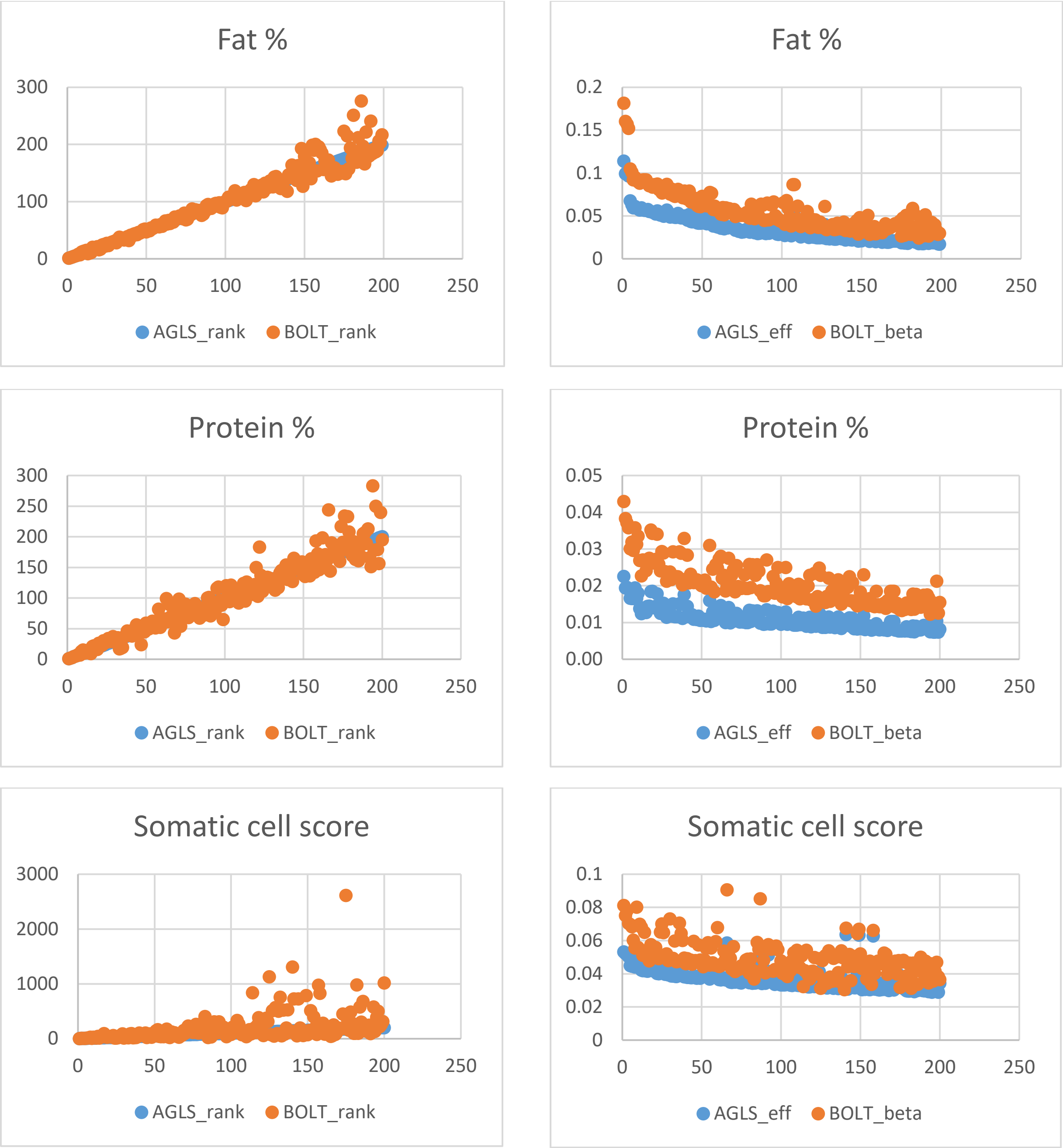

Fat %
300
200
100
0
0
50
100
150
200
250
AGLS_rank
BOLT_rank
Fat %
0.2
0.15
0.1
0.05
0
0
50
100
150
200
250
AGLS_eff
BOLT_beta
Protein %
300
250
200
150
100
50
0
0
50
100
150
200
250
AGLS_rank
BOLT_rank
Protein %
0.05
0.04
0.03
0.02
0.01
0.00
0
50
100
150
200
250
AGLS_eff
BOLT_beta
Somatic cell score
3000
2000
1000
0
0
50
100
150
200
250
AGLS_rank
BOLT_rank
Somatic cell score
0.1
0.08
0.06
0.04
0.02
0
0
50
100
150
200
250
AGLS_eff
BOLT_beta

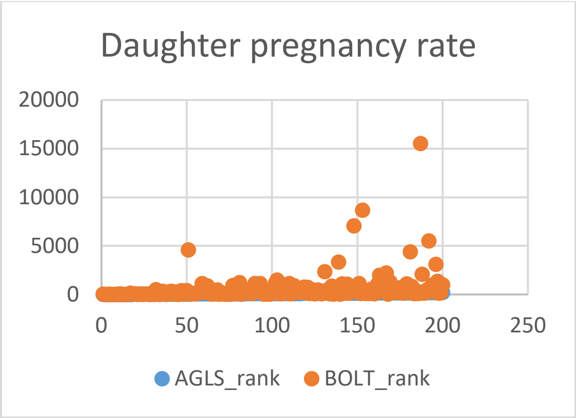
Daughter pregnancy rate
20000
15000
10000
5000
0
0
50
100
150
200
250
AGLS_rank
BOLT_rank

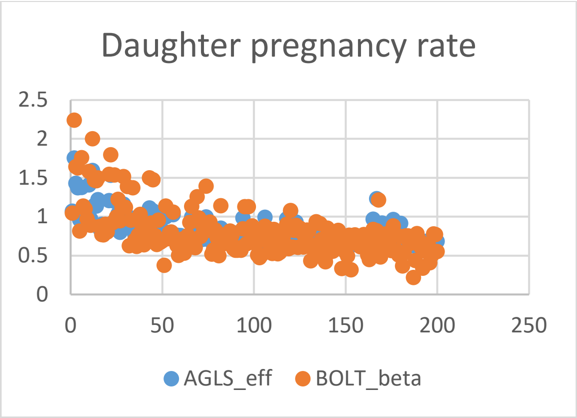
Daughter pregnancy rate
2.5
2
1.5
1
0.5
0
0
50
100
150
200
250
AGLS_eff
BOLT_beta

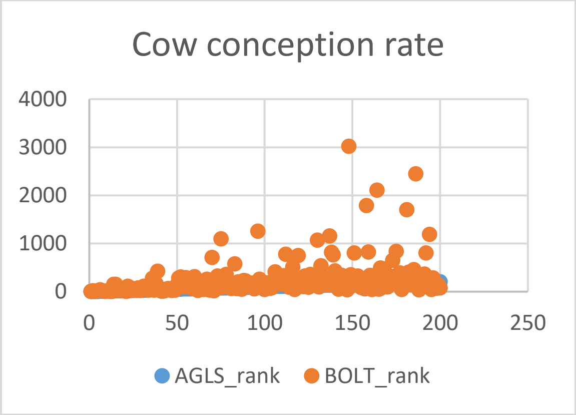
Cow conception rate
4000
3000
2000
1000
0
0
50
100
150
200
250
AGLS_rank
BOLT_rank

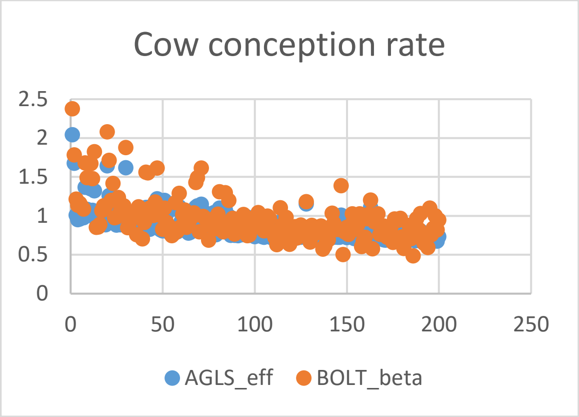
Cow conception rate
2.5
2
1.5
1
0.5
0
0
50
100
150
200
250
AGLS_eff
BOLT_beta

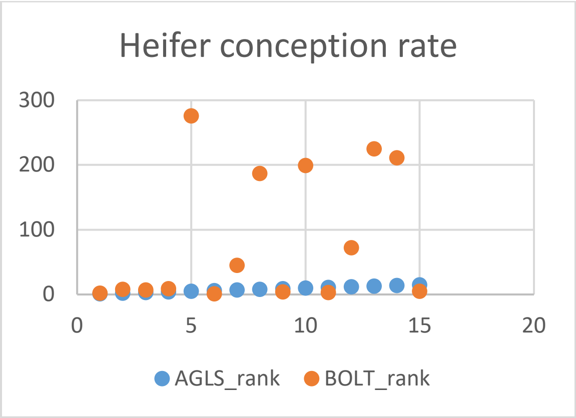
Heifer conception rate
300
200
100
0
0
5
10
15
20
AGLS_rank
BOLT_rank

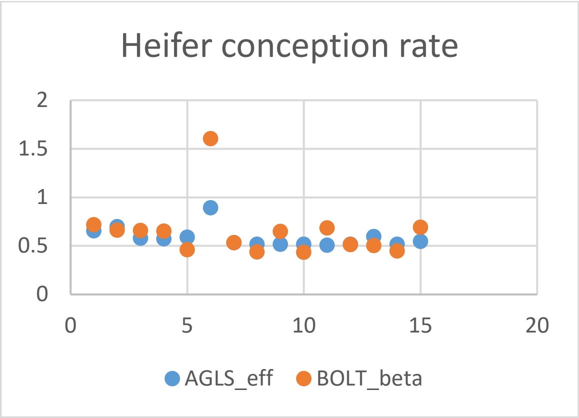
Heifer conception rate
2
1.5
1
0.5
0
0
5
10
15
20
AGLS_eff
BOLT_beta

FIGURE S4 | Comparison between statistical significance and allelic effects. The left column is the statistical significance of the additive effect of each SNP effects, and the right column is the allelic effects of each SNP. Fat and protein percentages are not included due to the complexity for interpreting those effects (see main text). For SCS, DPR, CCR and HCR, 'allelic mean' is used in place of 'allelic effect' by adding the common mean of all allelic means to each allelic effect, because the allelic effects for these traits were too small to display. A gene name (e.g., *DGAT1*) indicates the gene had at least one significant SNP effect, and a'-' between two genes indicates the SNP effect was between these two genes. 'd' indicates the significant SNP effect is downstream of the gene. 'u' indicates the significant SNP effect is upstream of the gene. SCS = somatic cell score, DPR = daughter pregnancy rate, CCR = cow conception rate, HCR = heifer conception rate.

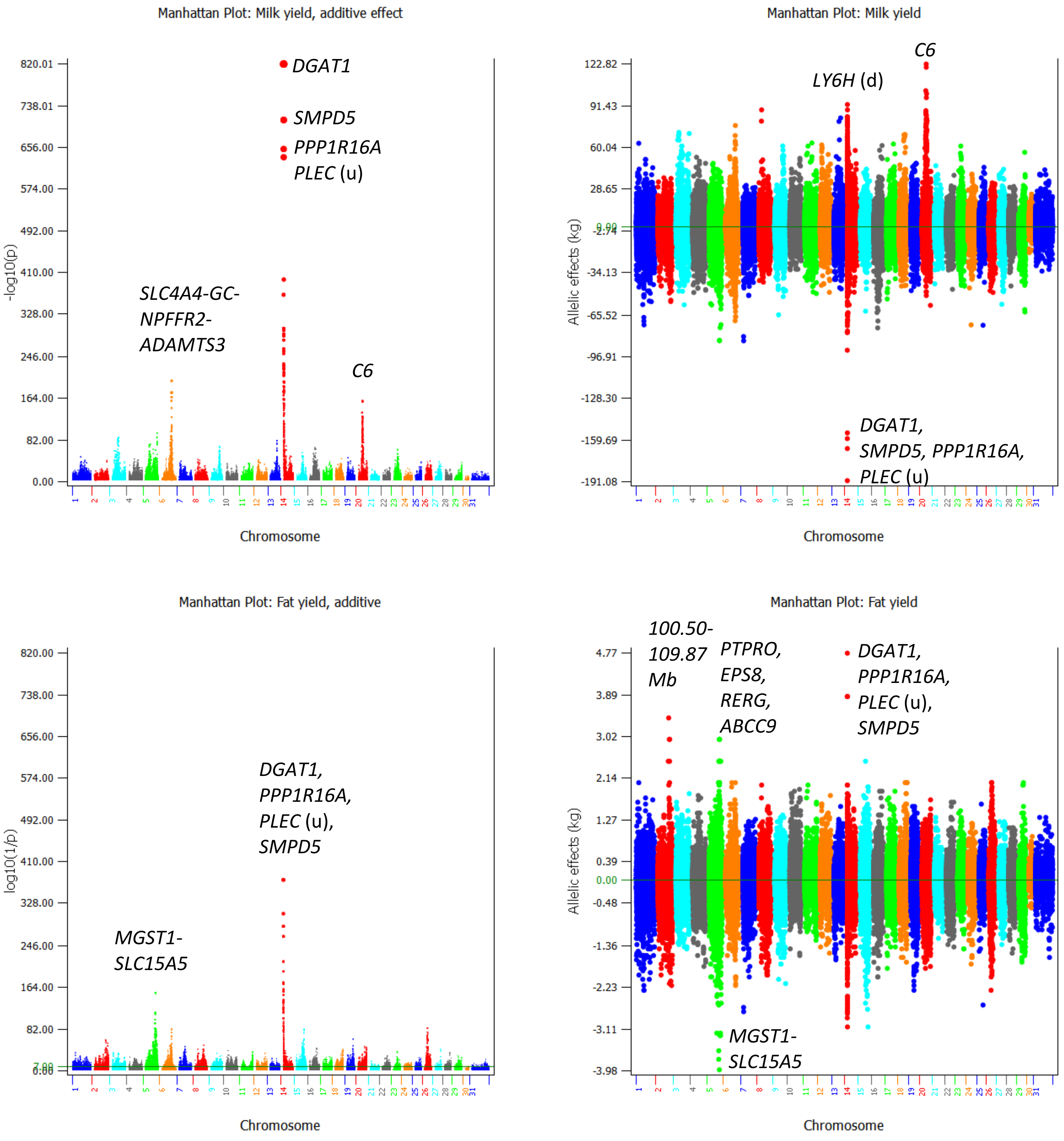

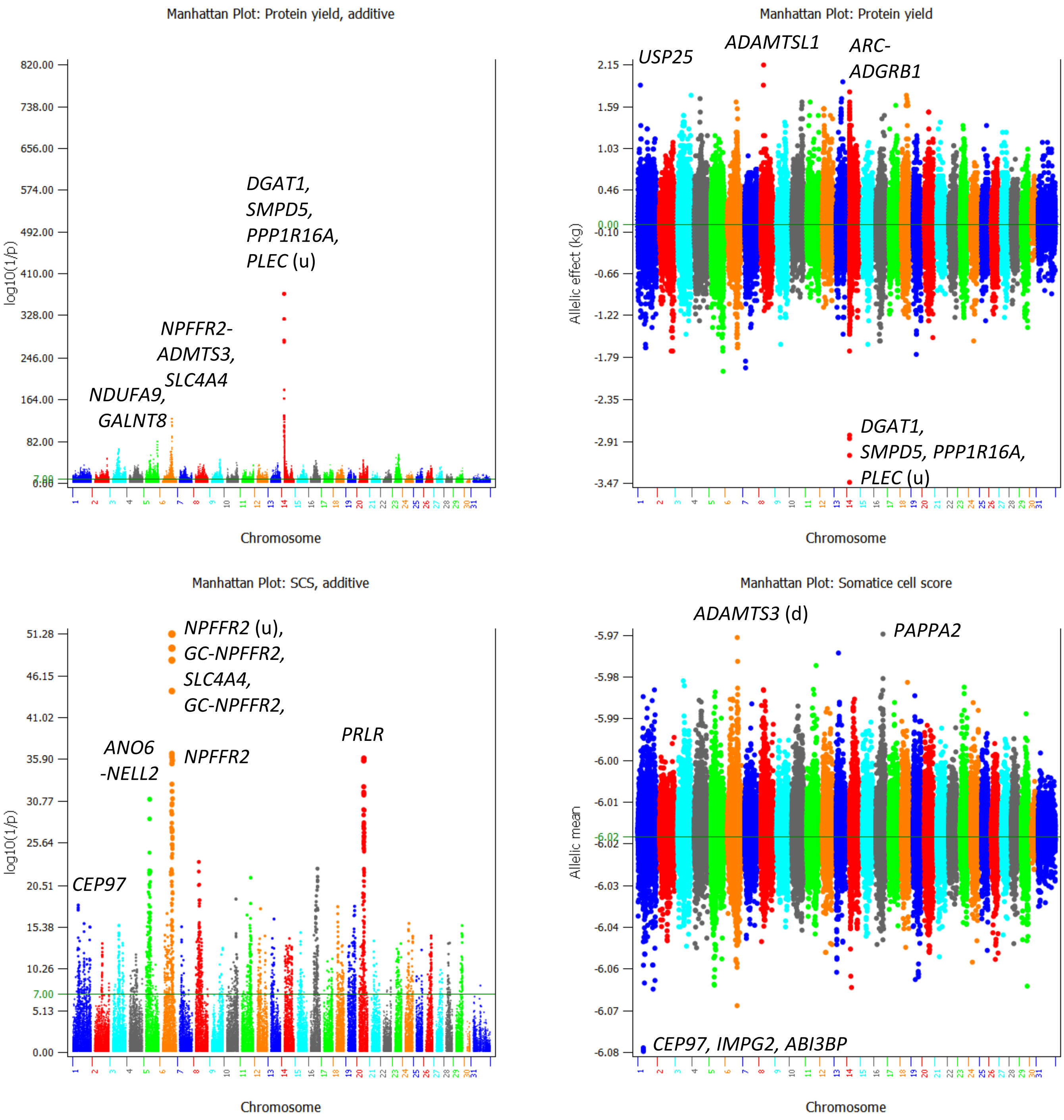
Manhattan Plot: Protein yield, additive
log10(1/p)
Chromosome
DGAT1,
SMPD5,
PPP1R16A,
PLEC (u)
NPFFR2-
ADMTS3,
SLC4A4
NDUFA9,
GALNT8
Manhattan Plot: Protein yield
Allelic effect (kg)
Chromosome
USP25
ADAMTSL1
ARC-
ADGRB1
DGAT1,
SMPD5, PPP1R16A,
PLEC (u)
Manhattan Plot: SCS, additive
log10(1/p)
Chromosome
NPFFR2 (u),
GC-NPFFR2,
SLC4A4,
GC-NPFFR2,
NPFFR2
ANO6
-NELL2
PRLR
CEP97
Manhattan Plot: Somatice cell score
Allelic mean
Chromosome
ADAMTS3 (d)
PAPPA2
CEP97, IMPG2, ABI3BP

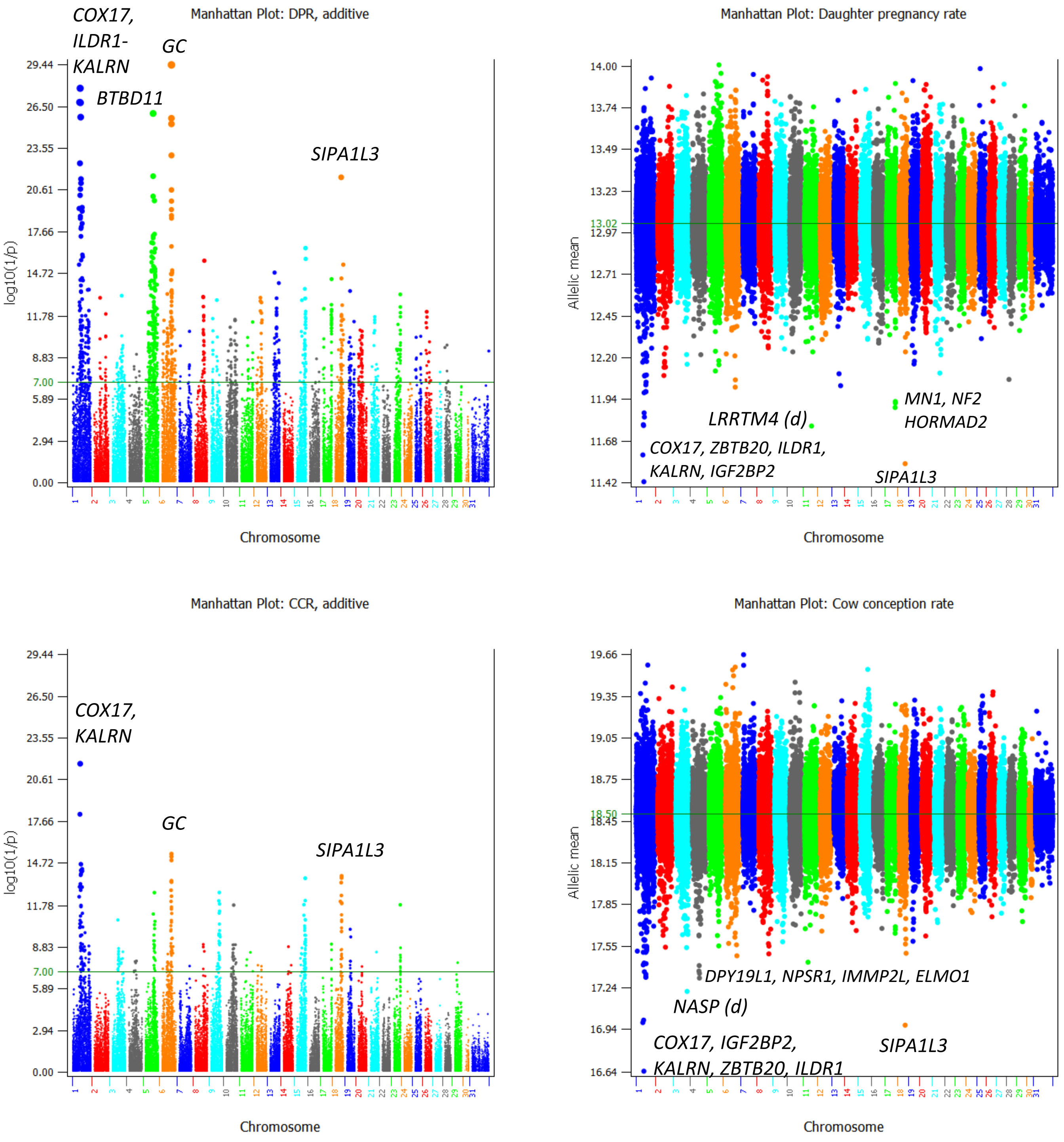
Manhattan Plot: DPR, additive
COX17, ILDR1-KALRN
GC
BTBD11
SIPA1L3
log10(1/p)
Chromosome
Manhattan Plot: Daughter pregnancy rate
Allelic mean
LRRTM4 (d)
MN1, NF2 HORMAD2
COX17, ZBTB20, ILDR1, KALRN, IGF2BP2
SIPA1L3
Chromosome
Manhattan Plot: CCR, additive
COX17, KALRN
GC
SIPA1L3
log10(1/p)
Chromosome
Manhattan Plot: Cow conception rate
Allelic mean
DPY19L1, NPSR1, IMMP2L, ELMO1
NASP (d)
COX17, IGF2BP2, KALRN, ZBTB20, ILDR1
SIPA1L3
Chromosome

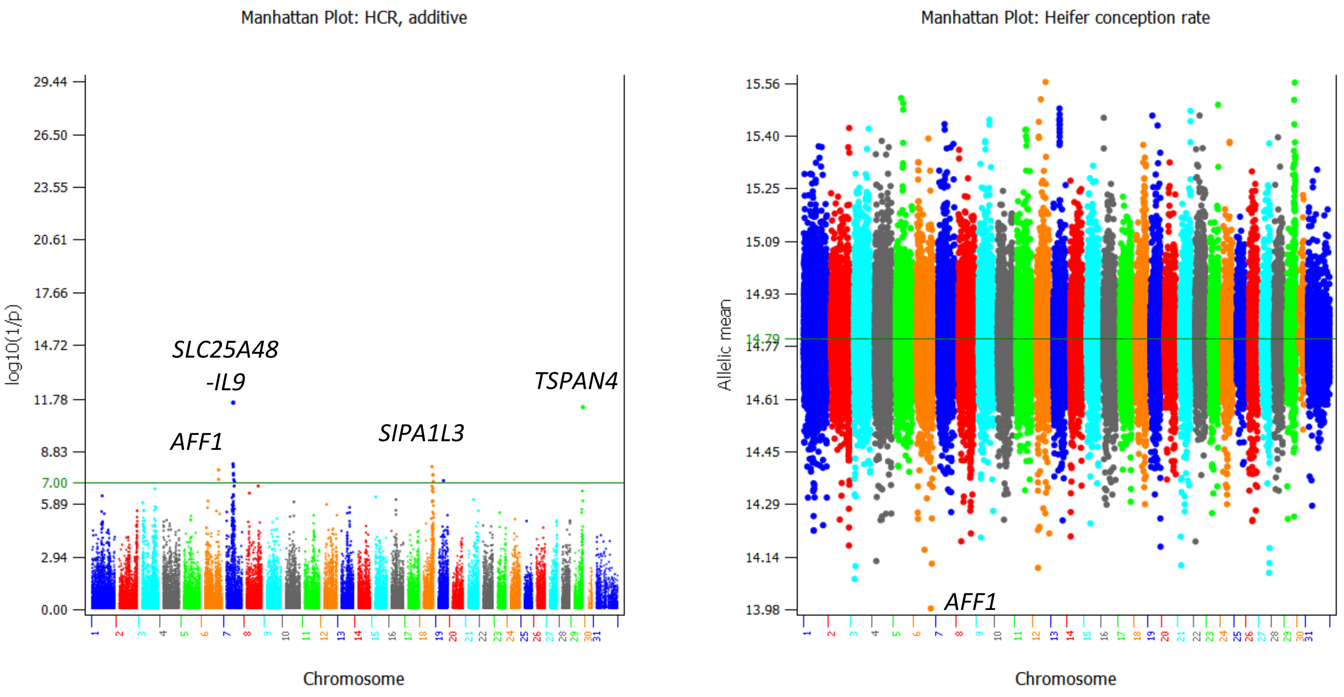

Manhattan Plot: HCR, additive
log10(1/p)
29.44
26.50
23.55
20.61
17.66
14.72
11.78
8.83
7.00
5.89
2.94
0.00
SLC25A48
-IL9
TSPAN4
AFF1
SIPA1L3
Chromosome
Manhattan Plot: Heifer conception rate
Allelic mean
15.56
15.40
15.25
15.09
14.93
14.79
14.77
14.61
14.45
14.29
14.14
13.98
AFF1
Chromosome

FIGURE S5 | The 2.08 Mb region of 1,379,063 to 3,464,083 bp of Chr14 containing *DGAT1* had nearly identical patterns of antagonism between fat yield and milk and protein yields. **A.** Antagonism between fat and milk yields, showing that the antagonism for these two traits was between the positive effects on fat yield and negative effects on milk yield indicated by the black line of (MY-)*(FY+). **B.** Antagonism between fat and protein yields, showing that the antagonism for these two traits was between the positive effects on fat yield and negative effects on protein yield indicated by the blue line of (PY-)*(FY+). The four genes are *DGAT1*, *PPP1R16A*, *SMPD5* and *PLEC* (upstream) had the strongest antagonistic pleiotropy. Antagonism also existed between negative fat effects and positive milk and protein effects but this antagonism, indicated by the red line of (MY+)*(FY-) and green line of (PY+)*(FY-), was much weaker than that indicated by the black and blue lines. (MY+) = positive effect on milk yield, (MY-) = negative effect on milk yield, (FY+) = positive effect on fat yield, (FY-) = negative effect on fat yield, (PY+) = positive effect on protein yield, and (PY-) = negative effect on protein yield.

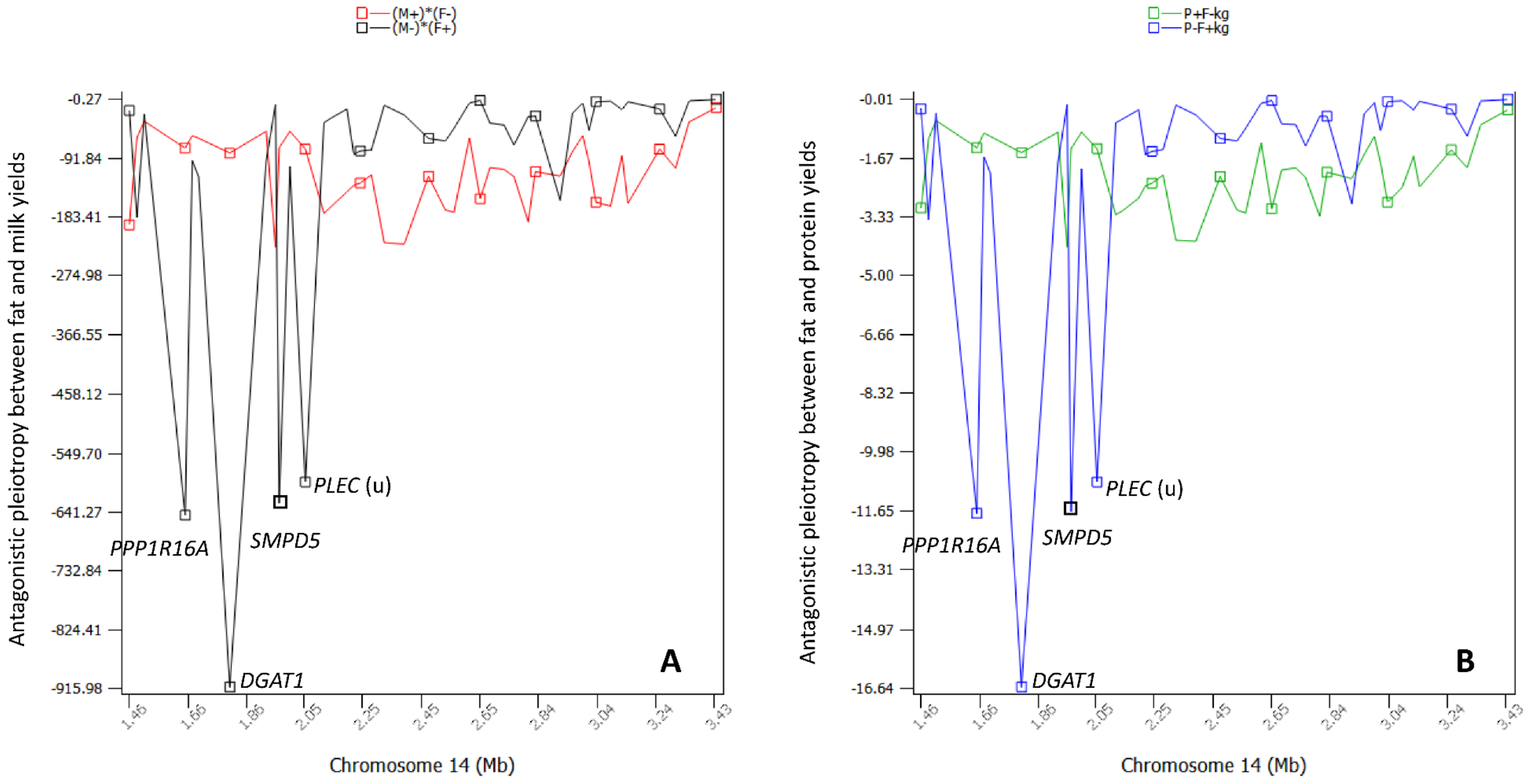

FIGURE S6 | The 58 years of genetic selection during 1957-215 in US Holstein cattle more than doubled milk yield from 5.3 tons in 1957 to 12.5 tons in 2015 based on the annual milk yields of 87,729,358 Holstein cows.

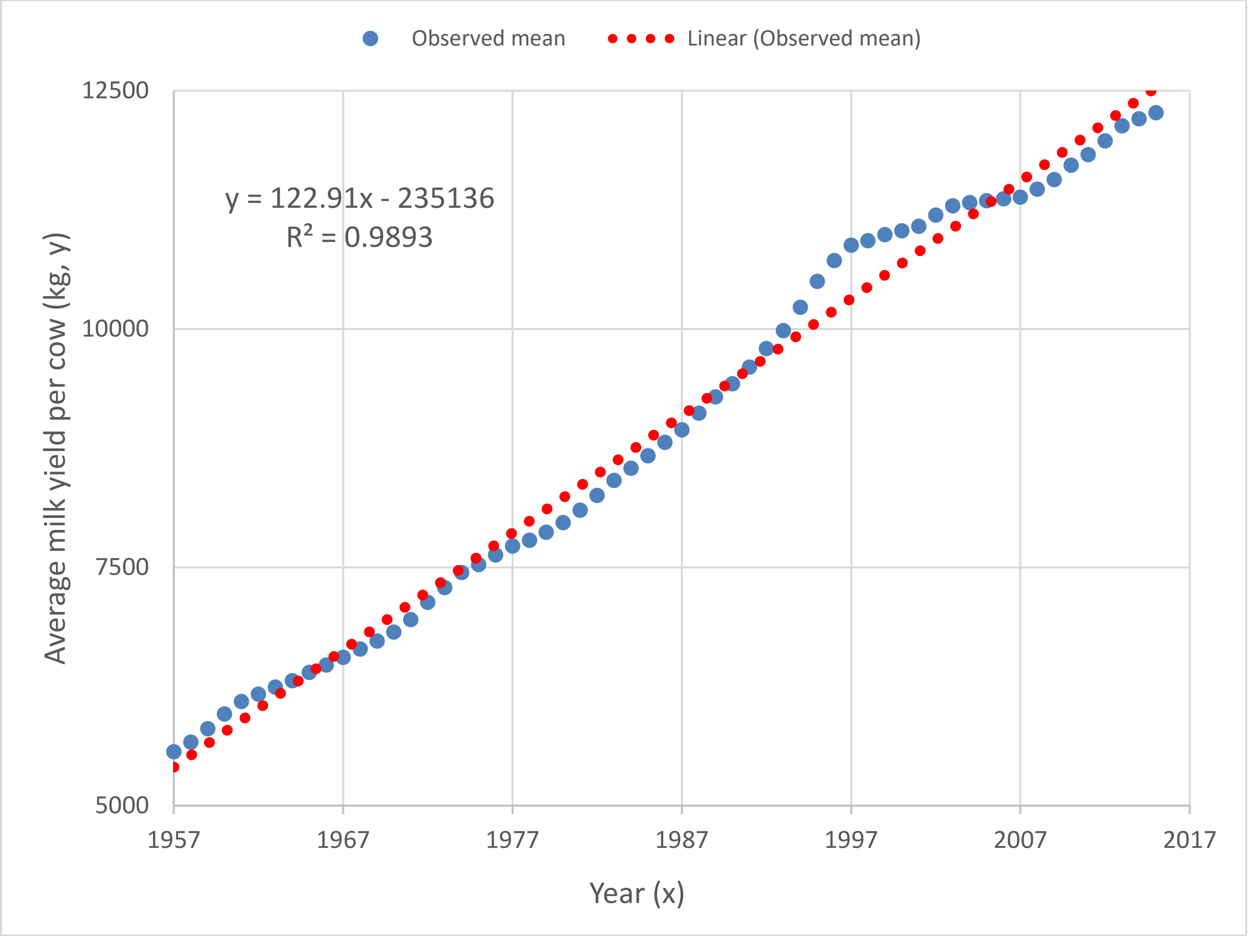

TABLE S1 | Number of observations for nine dairy traits.

MY	FY	PY	FPC	PPC	DPR	HCR	CCR	SCS
294,079	294,079	294,078	293,594	292,694	245,214	269,158	186,188	293,467

MY = milk yield. FY = fat yield. PY = protein yield. FPC = fat percentage. PPC = protein percentage. DPR = daughter pregnancy rate. CCR = cow conception rate. HCR = heifer conception rate. SCS = somatic cell score.

TABLE S2 | SNP chips for the GWAS population.

Chip name	No. SNPs	No. Cows
1	60671	5335
2	55660	583
3	43957	5302
4	43623	10789
5	43275	11555
6	39044	1125
7	38293	1195
8	15354	324
9	13137	17865
10	12545	4780
11	11626	50114
12	10619	48560
13	8426	31605
14	8051	23057
15	6987	462
16	6834	3563
17	6823	55677
18	2710	22188
Total		294,079

TABLE S3 | Birth years of the 294,079 cows for GWAS.

Birth year	Number of Cows	Total (percentage)
1983	1	
1987	2	
1989	4	
1990	4	
1991	15	
1992	28	
1993	28	
1994	61	
1995	52	
1996	95	
1997	112	
1998	81	
1999	114	
2000	119	
2001	185	
2002	303	
2003	502	
2004	1367	
2005	1659	4732 (1.6%)
2006	2252	
2007	3464	
2008	6037	
2009	10435	
2010	18105	
2011	33028	
2012	53328	
2013	82982	
2014	79647	
2015	69	289,374 (98.4%)

TABLE S4 | Number of SNP effects exceeding the statistical significance with the Bonferroni correction ($p<10^{-7}$) for nine dairy traits by AGLS and BOLT-LMM methods.

	MY	FY	PY	FPC	PPC	DPR	HCR	CCR	SCS	Total
					**AGLS**					
additive	11,856	9803	9984	11,349	15,215	112	15	360	2348	61,062
dominance	152	78	118	24	117	2	1	2	0	494
					**BOLT-LMM**					
additive	15,880	11,348	13,449	20,001	23,394	794	30	376	4203	89,457

MY = milk yield. FY = fat yield. PY = protein yield. FPC = fat percentage. PPC = protein percentage. DPR = daughter pregnancy rate. CCR = cow conception rate. HCR = heifer conception rate. SCS = somatic cell score.

TABLE S7 | Significant Chr14 additive effects after removal of *DGAT1* effects using AGLS method.

				DGAT1 effects removed			*DGAT1* effects not removed		
SNP	Chr	position (bp)	candidate gene	t-value	p-value	approximate rank [a]	t-value	p-value	rank
				**Milk yield**					
rs133739388	14	4870972	*KCNK9-COL22A1*	20.52	1.64E-93	119	12.66	1.07E-36	704
rs109208977	14	3649589	*GPR20* (d), duplication	19.85	1.31E-87	147	18.37	2.58E-75	188
rs110508680	14	4346983	*TRAPPC9*	19.78	5.18E-87	148	9.04	1.64E-19	2559
rs110090404	14	4103850	*PTK2-AGO2*	19.46	2.93E-84	149	20.98	1.12E-97	108
rs109752439	14	3985663	*PTK2*	19.41	7.02E-84	150	31.10	0.49E-211	28
				**Fat yield**					
rs135270011	14	2084067	*PLEC*	15.98	1.86E-57	123	31.20	9.84E-214	5
rs42304786	14	20831633	*SPIDR*	13.41	5.22E-41	255	8.23	1.84E-16	2678
rs41603869	14	1463676	*ZNF16 (d)*	13.18	1.20E-39	302	10.70	1.11E-26	810
rs41580044	14	7774329	*KHDRBS3 (d)*	13.09	3.76E-39	303	10.90	8.21E-28	738
rs137650898	14	2217163	*LOC506831*	12.96	2.04E-38	304	28.00	1.51E-172	7
rs42377728	14	20241456	blank	12.94	2.70E-38	305	10.70	9.22E-27	804
				**Protein yield**					
rs109225594	14	4848750	*KCNK9-COL22A1*	16.26	1.89E-59	81	22.30	109.49	24
rs109208977	14	3649589	*GPR20* (d), duplication	15.43	1.03E-53	99	14.20	44.79	149
rs110251237	14	4068825	*PTK2* (d)	15.05	3.89E-51	109	21.30	100.05	33
rs111018678	14	4336714	*TRAPPC9*	14.80	1.49E-49	121	20.50	92.75	37
rs41624797	14	3956956	*PTK2*	14.39	5.92E-47	135	22.20	108.51	25
rs136017274	14	4778358	*KCNK9* (d)	27.94	7.50E-42	170	18.60	76.37	54
				**Fat percentage**					
rs41624797	14	3956956	*PTK2*	46.08	1.0E-459	86	74.30	4.26E-1178	32
rs55617160	14	4468478	*TRAPPC9*	45.46	1.0E-447	89	73.40	4.48E-1150	33
rs111018678	14	4336714	*TRAPPC9*	44.67	1.0E-432	92	64.80	1.06E-900	47

rs110411273	14	3640788	duplication	44.54	1.0E-429	93	72.50	1.01E-1123	38
rs110185345	14	4043743	*PTK2*	41.83	1.0E-379	102	64.20	4.69E-883	48
rs109558046	14	2909929	*ARC-ADGRB1*	41.01	1.0E-365	106	108.00	4.84E-2463	5
**Protein percentage**									
rs109007040	14	67443766	*VPS13B*	57.39	1.0E-708	5	58.10	6.3E-725	5
rs136884351	14	67997855	*STK3* (d)	52.79	1.0E-601	8	8.91	5.28E-19	5353
rs137773528	14	65006380	*LOC104974118*	52.54	1.0E-595	9	53.60	1.4E-619	7
rs41632223	14	66494815	*RGS22*	52.13	1.0E-586	9	50.10	9.7E-541	16
rs41632193	14	66256529	*RNF19A*	49.54	1.0E-530	17	17.80	4.56E-71	809
rs41605240	14	69965123	*SDC2*	47.82	1.0E-494	22	49.00	6.0E-518	19

[a] This is the approximate ranking based on the comparison with similar p-value for SNP effect ranking without removing the *DGAT1* effects. 'u' indicates the SNP is upstream of the gene, and 'd' indicates the SNP is downstream of the gene. 'rank' is the rank of the statistical significance without removing the *DGAT1* effects. The duplication is at Chr14:2932132-3866447.
